# GLI2 inhibits cisplatin sensitivity in gastric cancer through DEC1/ZEB1 mediated EMT

**DOI:** 10.1038/s41419-025-07564-6

**Published:** 2025-03-25

**Authors:** Wenshuai Zhu, Jingguo Sun, Fubo Jing, Yuanxin Xing, Muhua Luan, Zhaotian Feng, Xiaoli Ma, Yunshan Wang, Yanfei Jia

**Affiliations:** 1 Research Center of Basic Medicine, Central Hospital Affiliated to Shandong First Medical University, Jinan, People’s Republic of China; 2 Research Center of Basic Medicine, Jinan Central Hospital, Shandong University, Jinan, People’s Republic of China; 3 Department of Medical Laboratory, Shandong Second Medical University, Weifang, People’s Republic of China

**Keywords:** Cancer therapeutic resistance, Oncogenes

## Abstract

Cisplatin (CDDP) based chemotherapy has emerged as the predominant therapeutic regimen for patients with advanced gastric cancer (GC). However, its efficacy is dampened by the development of chemoresistance, which results in poor prognosis of patients. GLI2, a key transcription factor in the Hedgehog (Hh) signaling pathway, is regarded as a target for cancer therapy. However, the significance of GLI2 for CDDP resistance in GC has not been well established. Here, we show that GLI2 expression was upregulated in EMT-type GC and associated with poor prognosis. GLI2 promotes proliferation, migration, and CDDP resistance of GC cells by inducing EMT. In terms of mechanism, GLI2 binds to the promoter region of DEC1 and enhances its expression, thereby co-transcriptionally regulating ZEB1 expression. Animal experiments have demonstrated that both GLI2 knockdown and GLI2 inhibitor significantly enhance CDDP sensitivity in GC. Our data not only identify a novel GLI2/DEC1/ZEB1/EMT pathway in GC CDDP resistance but also provide novel strategies to treat GC in the future.

## Introduction

Gastric cancer (GC) is a prevalent malignant tumor that affects the human digestive system, with China accounting for over 40% of new cases worldwide [1]. Diagnosis of GC often occurs at an advanced stage and despite surgical removal of the primary tumor and adjuvant chemotherapy, the 5-year survival rate remains low [2, 3]. Cisplatin (CDDP), as one of the first-line drugs, based on chemotherapy has emerged as the predominant therapeutic regimen for patients with advanced GC [2]. However, a significant percentage of cancer patients are inevitably resistant to CDDP. Herein, finding effective targets is imperative to explore the mechanisms underlying the progression of GC and thus be able to develop more efficient therapeutic strategies.

The Hedgehog (Hh) signaling pathway has been found to play an important and complex role in cancer progression [4, 5]. Glioma-associated oncogenes (GLIs) consist of three members: GLI1, GLI2, and GLI3, which are the downstream transcription factors involved in mediating the signaling pathway and leading to tumor progression [5, 6]. GLIs target genes have been linked to the promotion of numerous hallmarks of cancer, such as proliferation, survival, metastasis, and chemotherapeutic resistance [7–9]. Therefore, GLIs are regarded as the marker of abnormal activation of the Hh signaling pathway and may be a target for cancer therapy. Several lines of evidence indicate that activation of GLI transcription factors is required for oncogenesis in GC [10–12]. However, much emphasis has been placed on ligand-mediated activation of GLI1. The role of GLI2 transcription factors in regulating GC subtype identity and its downstream genes remains unclear.

Genomic and transcriptomic analyses have classified GC into molecular subtypes characterized by specific genetic aberrations and expression signatures that suggest important biological differences. Studies have shown that the epithelial- mesenchymal transition (EMT) molecular subtype was markedly associated with high genome integrity, poor survival, and drug resistance in GC [13, 14]. Some reports suggest that GLI1 can trigger EMT of cells by increasing the expression of Snail1 and vimentin but decreasing E-cadherin, acting as a transcription factor [15–17]. The zinc-finger transcription factor ZEB1 is most commonly characterized as an important driver of metastasis and therapy resistance through the induction of EMT [18–20]. Moreover, ZEB1 was reported to facilitate invasion and EMT, and upregulation of ZEB1 contributes to CDDP resistance in GC [21, 22]. GLI2 is an important player in the non-canonical Hh signaling pathway that operates independently of Smo activation. Recent studies have revealed that GLI2 influences the chemoresistance of GC cells by modulating tumor stem cell promotion [12, 23]. However, the significance of GLI2 for CDDP resistance in GC is not been well established.

Hence, this study suggested the need for a better understanding of the effects of GLI2 on EMT and CDDP resistance promotion in GC cells, and subsequent therapeutic studies. We show that GLI2 functions as a master regulator of the EMT subset of GC and define the DEC1/ZEB1 as a critical downstream target mediating this program. Moreover, our findings indicate that a GLI2/DEC1/ZEB1 axis can functionally substitute for CDDP sensitivity. Investigating the potential synergistic therapeutic value of combining GLI2 with CDDP treatment for GC provides a novel theoretical foundation and chemotherapy options for clinically resistant patients.

## Materials and methods

### Human tissue samples

A total of 101 tumor specimens from patients with GC were collected at the Central Hospital of Shandong First Medical University. These samples were collected consecutively from September 2018 to November 2021. The study was conducted in accordance with the guidelines approved by the Medical Ethics Committee of the Central Hospital of Shandong First Medical University. Informed consent was obtained from each patient participating in the study.

### Cell culture and reagents

Human gastric epithelial cells (GES1) and GC cell lines (MKN-45, HGC-27, MGC-803, AGS, and MKN-28) were obtained from the Shanghai Institute of Biochemistry and Cell Biology, Chinese Academy of Sciences. AGS cells were cultured in Ham’s F12 medium (MACGENE) and 10% FBS (Gibco). GES1 and other GC cell lines were maintained in RPMI-1640 (MACGENE) medium supplemented with 10% FBS. All these cells were cultured at 37 °C in a 5% CO_2_ humidified incubator (Thermo Fisher Scientific). All cell lines were routinely tested for mycoplasma infection. Short Tandem Repeat identification was performed on all cell lines.

### Cell transfection and lentivirus infection

The siRNA was purchased from Ribobio. The siRNA was transfected into cells using Lipofectamine 2000 and Opti-MEME transfection reagent. CRISPR-Cas9 and overexpression lentiviral vectors were constructed by GENECHEM. GC cells were inoculated in 24-well plates, cultured overnight, and then infected with lentivirus. The cells were subsequently incubated with puromycin (CWBIO) added to the medium for at least 1 week until there was no cell death.

### RNA isolation and real-time quantitative RT-qPCR

RNA was extracted using TRIzol reagent (CWBIO). RNA was reverse transcribed into cDNA using the HiFiScript gDNA Removal cDNA Synthesis Kit (CWBIO). RT-qPCR was performed on a LightCycler 480 Real-Time PCR System (Roche) using an UltraSYBR mixture (Low ROX; CWBIO). The sequences of primers are listed in Supplementary Table 1.

### Immunohistochemistry and H&E staining

Tumor tissues were fixed with 4% paraformaldehyde for 24 h and paraffin-embedded. Immunohistochemistry (IHC): 5 μm thick paraffin-embedded sections were deparaffinized twice with fresh xylene and then hydrated with gradient alcohol. Sections were subjected to heat exposure in EDTA antigen repair solution for 15 min. Sections were incubated with primary antibody overnight at 4 °C, then with biotin-labeled secondary antibody for 1 h at room temperature, and finally stained sequentially with 3,3′-diaminobenzidine tetrahydrochloride and hematoxylin. For the H&E staining, we used the H&E staining kit (Solarbio) to directly stain the nuclei and cytoplasm of cells. After the staining process, the tissue sections were gradually dehydrated with higher concentrations of ethanol and xylene. To preserve the stained samples, we sealed them with neutral resin.

### Western blot

Total protein was extracted with RIPA lysis buffer (CWBIO). The proteins were separated by 8% SDS-PAGE electrophoresis and then transferred to a PVDF membrane at 220 mA/2 h. After being closed with 5% skimmed milk for 2 h at room temperature, the membranes were hybridized with primary antibodies overnight at 4 °C. After hybridization, the membranes were incubated with HRP-coupled secondary antibodies for 45 min at room temperature and the bands were detected with ECL detection reagent (Millipore). Details of the primary antibodies and their respective dilutions can be found in Supplementary Table 2. Original data of western blot are reported as Original Data file.

### CCK-8 assay

Cell counts were determined using the Cell Counting Kit-8 (CCK-8, Elabscience). Briefly, cells were inoculated into 96-well plates and incubated with the indicated treatments. Subsequently, we added 60 µL of fresh medium to the cells containing 5 µL of CCK-8 solution and incubated them at 37 °C, 5% CO_2_ for 1 h. Then, the cell number was assessed by measuring the absorbance at 450 nm with a spectrophotometer.

### Ethynyldeoxyuridine (EdU) staining

Cell proliferation was assessed using the EdU staining assay. For the EdU assay (RiboBio), after inoculating cells into 96-well culture plates (5000 cells/well), cells were treated with EdU solution (100 μL/well) for 2 h, fixed with 4% paraformaldehyde, and then stained with Apollo and DAPI.

### Wound healing and transwell assays

For the wound healing assay, 2 × 10^5^ cells were inoculated into a 24-well plate until confluence. Cell monolayers were scratched using a pipette tip. Forty-eight or seventy-two hours later, the wounds were imaged using an inverted phase contrast microscope, the wound area was measured, and the percentage of wound closure was calculated.

For transwell assays, 5 × 10^4^ cells were suspended in 200 μL of low-serum medium and inoculated into 24-well upper chambers (8 μm pore size) with or without coating with matrix (BD Biosciences). The lower chamber was supplemented with 600 μL of medium containing 20% FBS, and after 48 or 72 h, cells affixed to the lower surface of the chamber were fixed with 4% paraformaldehyde, stained with 0.1% crystal violet, and counted under the microscope.

### Chromatin immunoprecipitation (ChIP) assay

The ChIP assay was performed according to the Cell Signaling Technology (CST) manufacturer’s protocol using the SimpleChIP® Plus Enzymatic Chromatin IP Kit (CST, 9002). Briefly, GC cells were fixed in 37% formaldehyde for 10 min at room temperature. Cross-linked chromatin DNA was sheared by sonication followed by immunoprecipitation using an anti-Flag antibody (CST). Normal rabbit IgG was included as a negative control. Chromatin Ab complexes were precipitated with magnetic beads and subsequently analyzed using quantitative qPCR. The primers used for ChIP-qPCR are listed in Supplementary Table 3.

### Dual-luciferase assay

Human ZEB1 Wild-type (WT) and mutant (MT) promoter reporters were cloned between the Renilla luciferase reporter gene and the Firefly luciferase reporter gene. pGL4.19 vector with wild-type promoter (WT) or mutated promoter sequences (MT) of ZEB1 respectively were transfected into HEK-293 T cells using Lipofectamine 2000 (Life Technologies, CA, USA). Luciferase activity was measured using the Dual- Luciferase Reporter Assay kit (Promega, USA) following the instructions and relative luciferase activity was normalized by Renilla luciferase activity. All transfection experiments were conducted in triplicate and repeated thrice independently.

### Co-immunoprecipitation (Co-IP)

Co-IP assays were performed using the Pierce® Immunoprecipitation Kit (Thermo Fisher Science) according to the manufacturer’s instructions. Briefly, MKN-45 cells were lysed with ice-cold IP lysis/washing buffer and fused by centrifugation at 13,000 × *g* for 10 min to remove debris. The supernatants were further immunoprecipitated with Flag antibody (CST). Rabbit IgG was used as a negative control. The precipitates were separated using SDS-PAGE and further analyzed by performing immunoblotting.

### Animal studies

We established a subcutaneous GC animal xenograft model to evaluate carcinogenicity in vivo. GC cells (7 × 10^6^) were injected subcutaneously into the axilla of 5-week-old BALB/c nude mice (Beijing Vitalever Laboratory Animal Technology), with 6 mice in each group. In addition, cisplatin (CDDP; 6 mg/kg, bid, 8 days) or GANT61 (50 mg/kg, bid, 8 days) was injected intraperitoneally into the nude mice in the additive group. Tumor growth was observed every 3 days and mice were culled after 21 days. The tumor volume was calculated as volume = (width^2^ × length × 0.5).

In addition, we established a tail vein metastasis model to assess metastatic properties. Similarly, 2 × 10^6^ cells were injected into the tail vein of nude mice. Mice were anesthetized with carbon dioxide and decapitated after 4 weeks, and then lung and liver tissues were removed and fixed with paraformaldehyde, followed by paraffin embedding. H&E staining was performed to observe tumor cell metastasis.

### Statistical analysis

GraphPad Prism 8.0 and R software (v4.1.3) were used for statistical analysis. Experiments were repeated independently at least three times. Student’s *t*-test one-way ANOVA was used to determine the significance of two and multiple groups, respectively. Two-way ANOVA was used to analyze the differences between the two groups over time. Spearman’s correlation was used to determine the expression correlation of the two genes. The chi-square test was used to determine the relationship between molecular and clinicopathologic variables. Mouse sample sizes were matched according to ANOVA degrees of freedom. Data are expressed as mean±mean square deviation. *P* < 0.05 statistically significant (ns, not significant, *P < 0.05, **P < 0.01, ***P < 0.001, ****P < 0.0001).

## Results

### GLI2 is upregulated in human GC and correlates with tumor progression

**Fig. 1:**
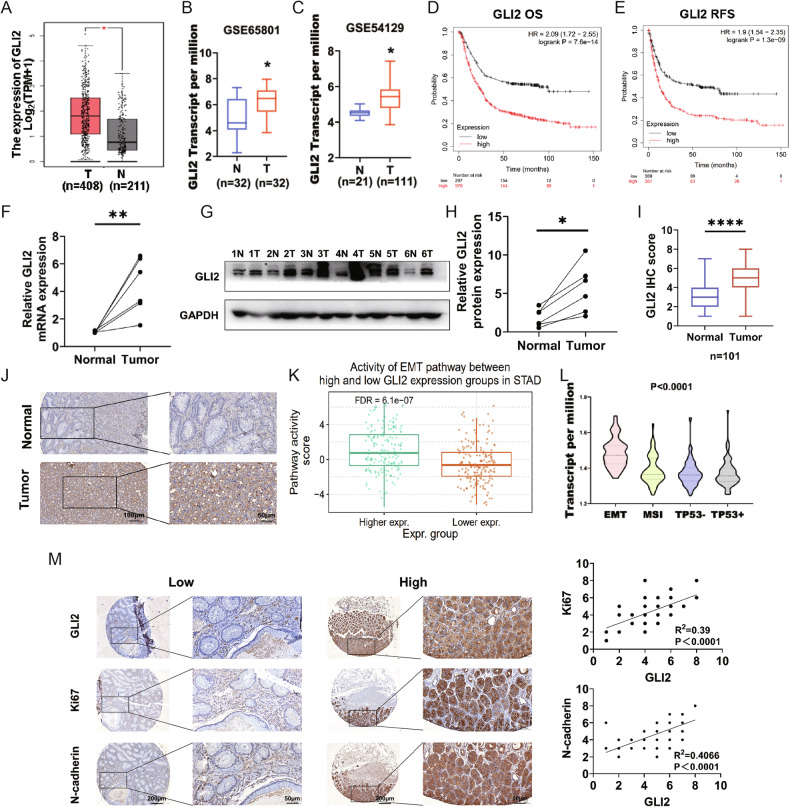
High expression levels of GLI2 were observed in the GC clinical samples and predicted poor prognosis. **A** The expression of GLI2 was significantly higher in 408 GC tissues than in 211 normal tissues. T tumor, N normal. **B**, **C** Comparing the expression level of GLI2 in GC tissues and normal gastric tissues using the GSE65801 and GSE54129 database. **D**, **E** Kaplan–Meier plotter analysis reveals that patients with higher GLI2 expression have shorter overall survival (OS) and recurrence-free survival (RFS) compared to patients with lower GLI2 expression. **F**–**H** GLI2 was overexpressed in the GC compared to expression in six pairs of corresponding adjacent normal tissues using qPCR and western blot. **I**, **J** IHC staining showed that the expression levels of GLI2 were significantly upregulated in clinical GC tissue samples. Scale bars: 100 µm (inset: 50 µm). **K**, **L** GLI2 is upregulated in both EMT subtype from the ACRG study and the EMT pathway activity from the TCGA project. **M** The representative IHC images of sections of GC samples showed Ki67 and N-cadherin expression in GC tissues with high or low GLI2 expression. ****P < 0.0001, **P < 0.01, *P < 0.05.

We explored the alteration in GLI2 abundance in GC tissues using The Cancer Genome Atlas Program (TCGA) and the Genotype-Tissue Expression (GTEx) database (http://gepia.cancer-pku.cn/). Our analysis revealed higher mRNA expression of GLI2 in GC tissues compared to normal gastric tissues (Fig. 1A). Publicly available transcriptomic datasets of GC tissues (GSE65801 and GSE54129) also indicated elevated GLI2 expression in GC tissues (Fig. 1B, C). Additionally, we utilized the Kaplan–Meier plotter (https://kmplot.com/ analysis), an online platform, to assess the overall survival (OS) and recurrence-free survival (RFS) of GC patients based on GLI2 mRNA expression level. The analysis revealed that patients with high GLI2 expression had shorter OS and PFS compared to patients with low GLI2 expression (Fig. 1D, E). Furthermore, we performed an additional verification using our own collection of clinical GC tissues, which further supported the higher expression of GLI2 in GC tissues compared to normal tissues (Fig. 1F–H). IHC analysis of 101 tissues from GC patients confirmed the high expression of GLI2 in GC tissues compared to normal tissues (Fig. 1I, J). Moreover, our analysis of data from clinical GC patients showed a significant correlation between high GLI2 expression and differentiation degree, pathological grade, lymph node metastasis, tumor size, and CDDP resistance (Table 1).

**Table 1:** Correlation between GLI2 and clinicopathologic parameters in gastric cancer.

Characteristic		Expression of GLI2		*X*^2^-test	*p* value
		High	Low		
Gender	Male	47	26	0.017	0.502
	Female	20	8		
Age (years)	≤50	57	30	0.45	0.897
	>50	10	4		
Pathological	Intestinal	6	3	0.04	0.98
	Diffuse	54	27		
	Signet ring	7	4		
Differentiation	G1 G2	15	19	11.33	0.001
	G3	52	15		
Max tumor diameter	>5 cm	47	16	5.12	0.024
	≤5 cm	20	18		
T classification	T1 T2	12	16	9.564	0.002
	T3 T4	55	18		
N classification	Positive	56	18	5.598	0.018
	Negative	11	16		
Clinical staging	I II	35	17	0.045	0.832
	III IV	32	17		
Cisplatin therapy	Sensitive	9	21	4.344	0.037
	Resistant	17	13		

GC has been classified based on molecular profiles, such as the Asian Cancer Research Group (ACRG) and TCGA. Molecular classifications were associated with different survival rates and recurrence patterns. GLI2 is upregulated in both EMT subtype from the ACRG study and EMT pathway activity from the TCGA project (Fig. 1K, L). EMT has been previously linked to increased tumor-initiating, metastatic potential, and therapeutic resistance [24]. We further measure EMT, cell proliferation markers and GLI2 expression levels using clinical tissue samples. The results indicated a positive correlation between GLI2 expression and the expression of Ki67 and N-cadherin (Fig. 1M). These findings suggest that GLI2 may play a crucial role in the development of GC.

### GLI2 depresses CDDP sensitivity and promotes metastasis via EMT in vitro

**Fig. 2:**
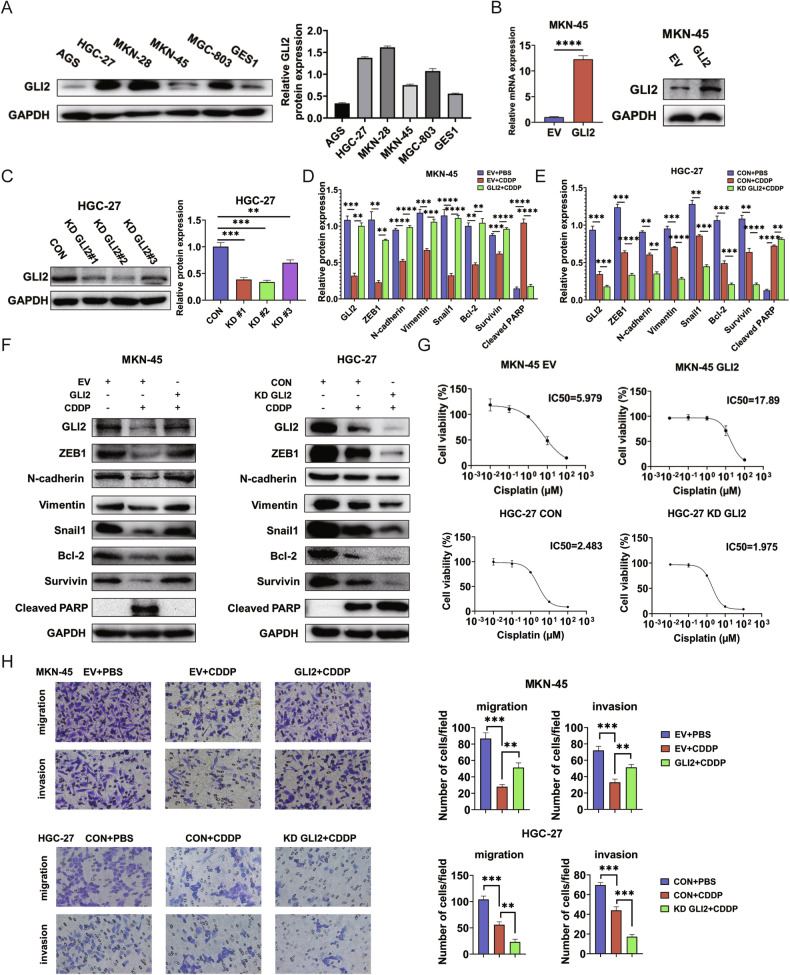
GLI2 depresses CDDP sensitivity and promotes GC cell metastasis via EMT in vitro. **A** Western blot analysis of GLI2 expression in GC cell lines and normal gastric epithelial cells. **B**, **C** MKN-45 cells with stable GLI2 overexpression or HGC-27 cells with GLI2 knockdown were created. The changes in GLI2 expression were confirmed using qPCR or western blot. **D**–**F** Western blot analysis investigated the effect of GLI2 on the expression of indicated proteins combined with CDDP treatment. **G** CCK-8 experiment shows the IC50 values for CDDP in indicated cells. **H** Transwell experiment tested the impact of GLI2 on the migratory and invasion ability of GC cells under CDDP treatment. Scale bars: 50 µm. ****P < 0.0001, ***P < 0.001, **P < 0.01, *P < 0.05.

In human cancer, EMT is a key cell process associated with tumor progression and resistance to therapy. Therefore, we assessed the effect of GLI2 on the EMT of GC cells. First, we analyzed the expression levels of GLI2 in these cells using western blot analysis. The results showed that the expression of GLI2 was significantly higher in GC cells (HGC-27, MKN-28, and MGC-803) compared to normal gastric mucosal epithelial cells GES1 (Fig. 2A). To further understand the impact of GLI2 on GC cell behavior, We created stable GLI2 overexpression in MKN-45 and GLI2 knockdown (KD) cell lines in HGC-27 using CRISPR-Cas9 technology (Fig. 2B, C). We selected KD GLI2#2 for further investigation. To assess the effects of GLI2 on cell behavior, we evaluated proliferation and migration abilities using EdU incorporation, transwell assays, and wound healing experiments. The results revealed that overexpression of GLI2 promoted the proliferation and migration of GC cells, while knockdown of GLI2 inhibited these cellular processes (Supplementary Fig. 1A–E). We then detected the expression level of EMT biomarkers (ZEB1, N-cadherin, Vimentin, and Snail1) (Supplementary Fig. 1F). Importantly, GLI2 overexpression significantly antagonized CDDP downregulated expression of these mesenchymal phenotypic markers and cell survival, while GLI2 silencing had the opposite effect (Fig. 2D–F). Cells were also treated with GANT61, a GLI antagonist, or DMSO. The combination of CDDP and GANT61 also resulted in downregulated expression of these mesenchymal phenotypic markers and cell survival (Supplementary Fig. 1G). We also found GLI2 promoted CDDP resistance and EMT in MKN-28 cells (Supplementary Fig. 1H, I). CDDP or GANT61 alone can suppress GC cells growth, and the combination produces an apparent decrease in cell proliferation (Supplementary Fig. 2A). Therefore, we hypothesized that GLI2 exerts an anti-resistance function by regulating EMT. Moreover, we found the IC50 value of CDDP for GLI2 overexpression in MKN-45 cells is higher than control groups whereas GLI2 knockdown in HGC-27 decreased the IC50 value of CDDP (Fig. 2G). In the transwell assay, GLI2 overexpression decreased while GLI2-KD increased CDDP-inhibited migration and invasion (Fig. 2H). We further found CDDP could decrease the protein stability of GLI2 (Supplementary Fig. 2B, C). We used SUnSET method to validate the effect of GLI2 on protein synthesis. We observed a significant decrease in puromycin uptake when GLI2 was silenced (Supplementary Fig. 2D). These results indicate that GLI2 can regulate the sensitivity of GC cells to CDDP by affecting the process of EMT.

### GLI2 inhibits CDDP sensitivity in vivo

**Fig. 3:**
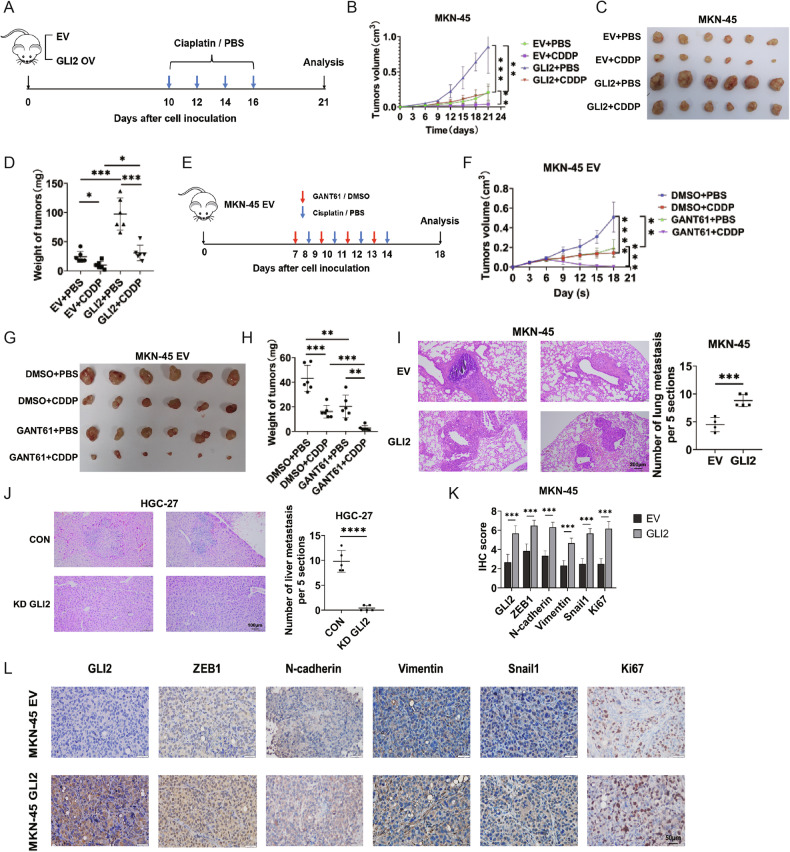
GLI2 inhibits CDDP sensitivity in vivo. **A** Schematic description of the experimental design for establishing the GLI2 overexpression animal model. **B**–**D** Representative images of tumors, tumor growth curves, and tumor weight in the xenograft model. **E** Schematic description of the experimental design for establishing the GANT61 treated animal model. **F**–**H** Representative images of tumors, tumor growth curves, and tumor weight in the xenograft model. **I**, **J** Representative images of lung (**I** scale bars = 200 μm) and liver (**J** scale bars = 100 μm) metastasis and H&E staining are shown. **K**, **L** IHC staining showed GLI2, ZEB1, N-cadherin, Vimentin, Snail1, and Ki67 expression in xenografts. Scale bars: 50 μm. ****P < 0.0001, ***P < 0.001, **P < 0.01, *P < 0.05.

Next, we explored the role of GLI2 in CDDP sensitivity of GC in vivo. Animal experiments were performed according to the flowchart as shown in Fig. 3A. The results demonstrated that the tumors in the GLI2-overexpressing group exhibited significantly accelerated growth compared to those in the control group (Fig. 3B–D). Intraperitoneal administration of CDDP effectively suppressed the tumor growth rate and reduced subcutaneous tumor volume. However, when combined with GLI2 overexpression in the CDDP-treated group, there was partial restoration observed in terms of tumor volume and growth rate (Fig. 3B–D). Consistently, GANT61, a confirmed inhibitor of GLI, effectively suppresses the in vivo expression of GLI2 and markedly reduces tumor volume and weight (Fig. 3E–H). Treatment with GANT61 together with CDDP conferred the highest inhibition on tumor growth (Fig. 3E–H). Furthermore, there is a significant increase in lung nodules in the MKN-45 group with GLI2 overexpression compared to the control group, while liver nodules were significantly reduced in the HGC-27 group with GLI2 knockdown compared to the control group (Fig. 3I, J). These findings indicate that GLI2 significantly enhances the ability of GC cells to metastasize. To further validate GLI2 induction of EMT, IHC was performed on isolated mouse tumors. Consistently, GLI2 overexpression increased ZEB1, N-cadherin, Vimentin, Snail1, and Ki67 levels (Fig. 3K, L). This finding suggests that GLI2 inhibits CDDP sensitivity by promoting the EMT of GC in vivo.

### GLI2 activates the transcription of DEC1 in GC

**Fig. 4:**
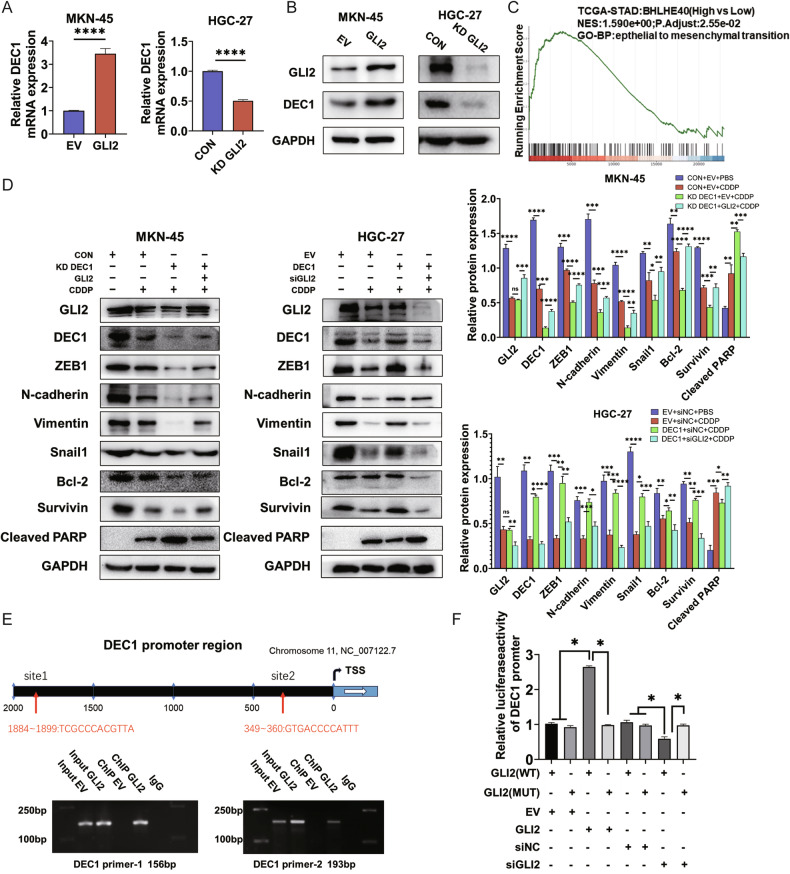
GLI2 activates the transcription of DEC1 in GC. **A**, **B** The mRNA and protein expression of GLI2 and DEC1 were detected by qRT-PCR and western blot. **C** GSEA was conducted using the TCGA-STAD data to determine the correlation between DEC1 and EMT via the CAMOIP online tool. **D** Western blot analysis investigated the effect of GLI2/DEC1 joint regulation for the indicated groups. **E** DEC1-promoter binding sites predicted by online site JASPAR, validated by ChIP. **F** Transcriptional activity of DEC1 was measured by the luciferase reporter system. Reporter activity is reported as the fold activation relative to Renilla luciferase activity. ****P < 0.0001, ***P < 0.001, **P < 0.01, *P < 0.05.

RNA-Seq analysis was then conducted to examine gene expression in EV and GLI2-overexpressing MKN-45 cells and investigate the underlying mechanisms revealing the oncogenic potential of GLI2 in GC cells. We found GLI2 overexpression significantly upregulated DEC1 expression (Supplementary Fig. 3A). Correlation analysis in the TCGA database also showed that the expression level of DEC1 was significantly associated with GLI2 (Supplementary Fig. 3B). PCR and Western blot analysis confirmed that overexpression of GLI2 significantly promoted DEC1 mRNA and protein levels in GC cells, while GLI2 knockdown reduced them (Fig. 4A, B). IHC detection of tumor tissue from BALB/c Nude mice further validated the in vivo regulation of DEC1 expression by GLI2 (Supplementary Fig. 3C). GSEA was conducted using the TCGA-STAD data to determine the correlation between DEC1 and EMT via the CAMOIP online tool (http://camoip.net/). Consequently, EMT was enriched in the high DEC1 expression group (*P* < 0.05, Fig. 4C). Thus, we hypothesize that GLI2 activates DEC1 transcription and then contributes to EMT and chemotherapy resistance in GC. Subsequently, to determine whether DEC1 is indispensable for GLI2-mediated EMT and CDDP sensitivity, lentivirus-mediated stable knockdown of DEC1 was established in MKN-45 cells, while lentivirus-mediated stable overexpression of DEC1 was achieved in HGC-27 cells (Supplementary Fig. 3D). As expected, DEC1 KD reversed the GLI2-mediated upregulation of mesenchymal phenotype marker (ZEB1, N-cadherin, Vimentin, Snail1) and anti-apoptosis molecular (Bcl-2, Survivin) (Fig. 4D, Supplementary Fig. 3E). Notably, the induced CDDP-mediated EMT and apoptosis by GLI2 overexpression were markedly suppressed by DEC1 knockdown, while DEC1 overexpression had the reverse effect (Fig. 4D). ChIP assays verified GLI2 transcriptionally activates DEC1 through direct binding to its promoter (Fig. 4E). The dual-luciferase assay was conducted according to the binding site (Fig. 4F) and the co-transfection of GLI2 overexpression and DEC1-promoter WT markedly induced the luciferase activity, while GLI2 silencing had the opposite effect, indicating the targeted binding between GLI2 and DEC1 (Fig. 4F). Thus, GLI2 transcriptionally regulates DEC1 and enhances its expression in GC cells.

### DEC1 is essential for GLI2-mediated CDDP resistance

**Fig. 5:**
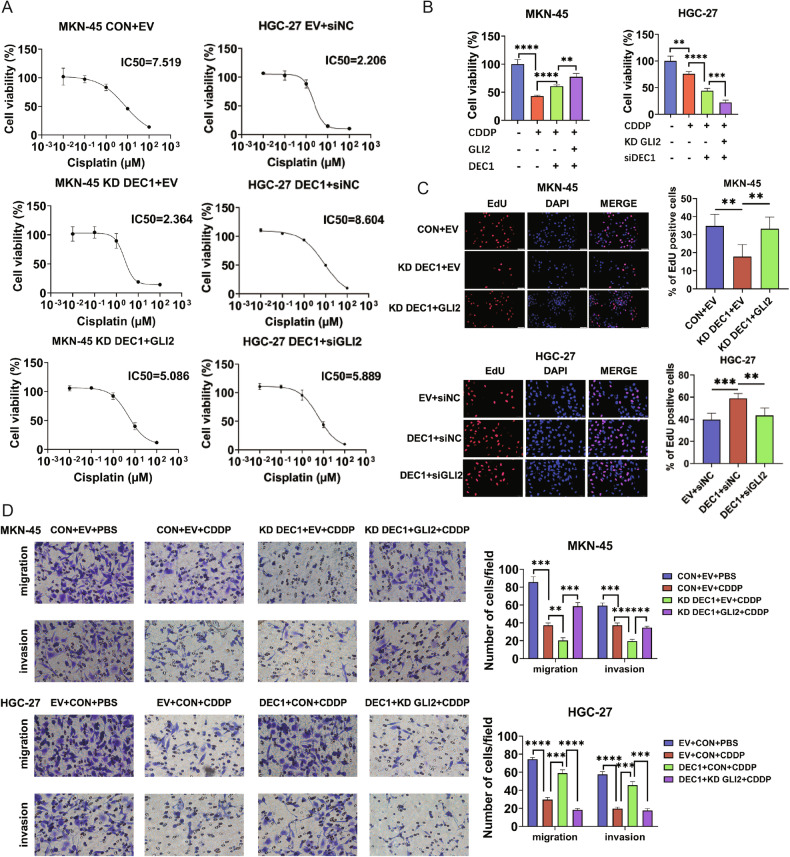
DEC1 is essential for GLI2-mediated CDDP resistance. **A** CCK-8 experiment shows the IC50 values for CDDP with GLI2 and DEC1 co-expression alteration in GC cells. **B** Cell viability of GC cells with GLI2 and DEC1 overexpression or knockdown under CDDP treatment was determined by CCK-8 assays. **C** EdU incorporation assays measured the effects of GLI2 and DEC1 on cell proliferation. Scale bars are 50 μm. **D** Transwell experiment tested the impact of GLI2 and DEC1 on the migratory and invasion ability of GC cells under CDDP treatment. Scale bars: 50 µm. ****P < 0.0001, ***P < 0.001, **P < 0.01, *P < 0.05.

Through cellular functional experiments, we discovered that DEC1 is indispensable for GLI2-mediated CDDP resistance. DEC1 knockdown abolished the cell resistance of GC to CDDP caused by GLI2 overexpression (Fig. 5A). In addition, DEC1 overexpression reversed the GC cell sensitivity to CDDP caused by low GLI2 expression (Fig. 5A). We utilized the Kaplan–Meier plotter, an online platform to assess the RFS of GC patients based on DEC1 mRNA expression level. The results also demonstrated patients with higher DEC1 expression have shorter RFS compared with patients with lower expression (Supplementary Fig. 4A). Furthermore, we examined the Cancer Therapeutics Response Portal (CTRP) datasets [25] (http://www.broadinstitute.org/ctrp/) and assessed associations between GLI2 and DEC1 expression data across GC cell lines and cell sensitivities to CDDP. The results showed that the expression of GLI2 and DEC1 was negatively correlated with the sensitivity of GC cells to the CDDP (Supplementary Fig. 4B), suggesting that GC cells with high GLI2 expression are resistant to CDDP. We also found synergistic function of GLI2 and DEC1 on sensitization to CDDP and EMT (Fig. 5B and Supplementary Fig. 5A). EdU assays confirmed that knockdown of DEC1 reduced the proliferative capacity of GC cells, while co-overexpression of GLI2 reversed this weakened proliferation ability (Fig. 5C). Transwell and scratch assays revealed that after suppression of DEC1 expression, the induction of cell migratory ability caused by GLI2 overexpression was largely restored, whereas DEC1 overexpression simultaneously reversed the GLI2-reduced migratory ability of GC cells (Supplementary Fig. 5B, D). We also proved that DEC1 knockdown suppressed CDDP-mediated cell migration and invasion by GLI2 overexpression, while DEC1 overexpression had the opposite effect (Fig. 5D).

**Fig. 6:**
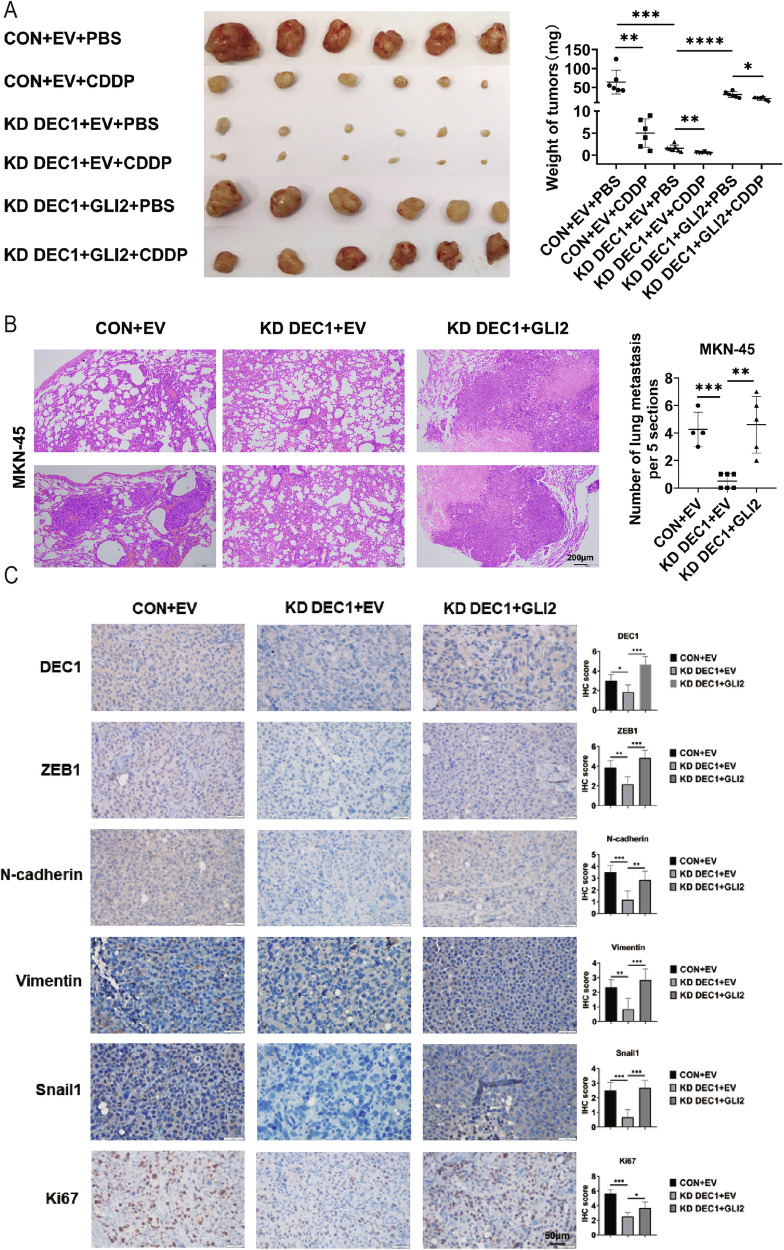
GLI2 and DEC1 co-regulate tumor growth, metastasis, and CDDP sensitively in vivo. **A** Subcutaneous tumor models (*n* = 6 mice per group) were established using stable expressed MKN-45 cells combined with CDDP intraperitoneal injection. Tumor weights were analyzed. **B** Representative H&E images (scale bar: 200 μm) of lung samples from the indicated groups of nude mice. **C** IHC staining for DEC1 ZEB1, N-cadherin, Vimentin, Snail1, Ki67 expression in xenografts. Scale bars: 50 μm. ***P < 0.001, **P < 0.01, *P < 0.05.

To investigate whether the GLI2/DEC1 axis is essential for tumor formation in vivo, we isolated tumors from mice. In the subcutaneous tumor model of DEC1 KD MKN-45 cells, CDDP-inhibited GC tumor growth more efficiently than DEC1 CON cells or CDDP treatment alone (Fig. 6A). However, for DEC1 KD MKN-45 cells transfected with GLI2, CDDP-inhibited GC tumor growth less efficiently than the DEC1 KD MKN-45 group (Fig. 6A). Meanwhile, by quantifying the number of metastases in the lung, we observed a significant decrease in tumor metastasis burden in the DEC1 KD group compared to the control group. However, simultaneous overexpression of GLI2 in the DEC1 KD group partially restores lung metastases compared to DEC1 KD treatment alone (Fig. 6B). IHC results obtained from xenograft tumor sections showed decreased levels of DEC1, ZEB1, N-cadherin, Vimentin, Snail1 and Ki67 in DEC1 KD tumor tissue compared to control sections (Fig. 6C). Simultaneous overexpression of GLI2 in the DEC1 KD group partially restored the proportion of DEC1, ZEB1, N-cadherin, Vimentin, Snail1, and Ki67-positive cells (Fig. 6C) Those results suggest that DEC1 is essential for GLI2-mediated CDDP resistance.

### DEC1 promotes EMT and CDDP resistance by targeting ZEB1

**Fig. 7:**
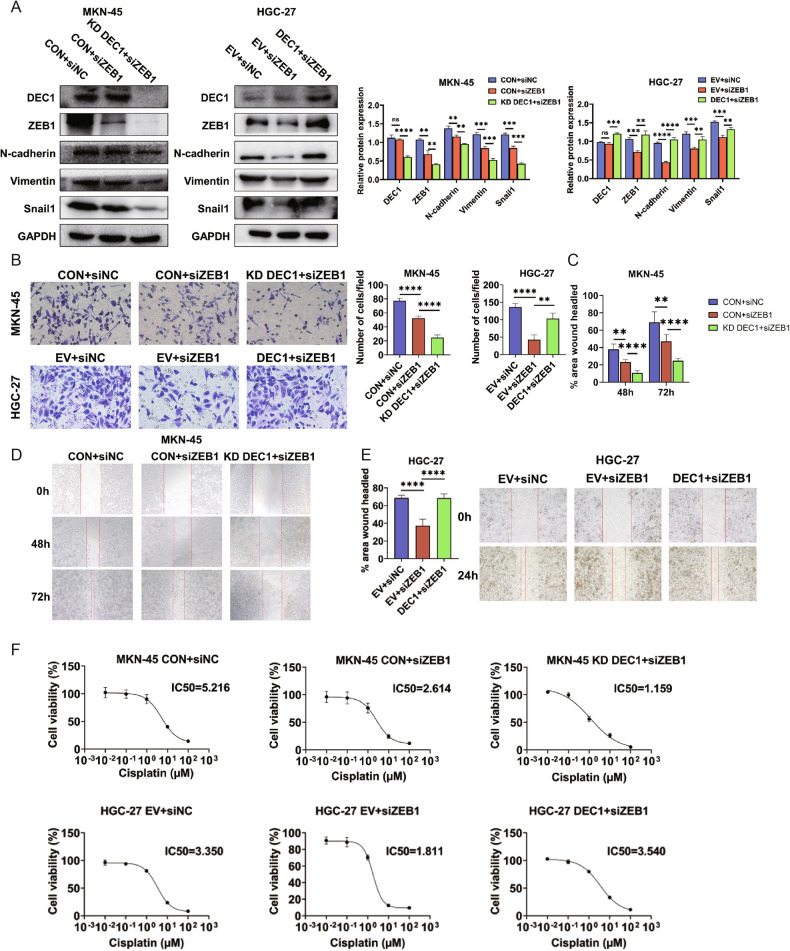
DEC1 promotes EMT and CDDP resistance by targeting ZEB1. **A** Western blot analysis investigated the effect of DEC1 and ZEB1 joint regulation on ZEB1, N-cadherin, Vimentin, Snail1 expressions. **B**–**E** The effect of DEC1 and ZEB1 on the migration of GC cells was analyzed by transwell assay and wound healing assay. Scale bars:200 µm. **F** CCK-8 experiment shows the IC50 values for CDDP with DEC1 and ZEB1 co-expression alteration in GC cells. ****P < 0.0001, ***P < 0.001, **P < 0.01, *P < 0.05.

The EMT process has been proposed to mediate chemoresistance. To identify the potential EMT molecules involved in GLI2/DEC1 axis-mediated CDDP resistance in GC, the correlation between the expression of EMT molecules and DEC1 was calculated using PCR. The results showed that ZEB1 had a positive correlation with DEC1 in MKN-45 cells (Supplementary Fig. 6A). Silencing ZEB1 could further inhibit the EMT process and cell migration in DEC1 KD cells (Fig. 7A; Supplementary Fig. 6B, C). Moreover, inhibition of ZEB1 effectively counteracted the DEC1-induced EMT process and cell migration (Fig. 7A–E). ZEB1 was strongly correlated with CDDP-resistance in CTRP (Supplementary Fig. 4B). CCK-8 assays also showed inhibition of ZEB1 exhibited a sensitizing effect toward CDDP. This effect could be enhanced by DEC1 KD, while alleviated by overexpression of DEC1 (Fig. 7F). We also generated CDDP-resistant MKN-45 cells following multiple treatments with CDDP. A cell viability assay was utilized to assess the acquired CDDP resistance. The IC50 for CDDP in the resistant MKN-45 cells (named MKN-45 R) is 25.27 µM whereas that of the parental cells is only 7.91 µM (supplementary Fig. 7A). Next, we compared protein expression of EMT pathways. We found GLI2 overexpression significantly increased the expression of MRP1, DEC1, and mesenchymal phenotype markers (ZEB1, N-cadherin, Vimentin, and Snail1). Knockdown GLI2 in CDDP-resistant MKN-45 cells inhibited mesenchymal phenotype molecular expression (supplementary Fig. 7B, C).

### GLI2 and DEC1 form a protein complex that can cooperatively transactivate ZEB1 expression

**Fig. 8:**
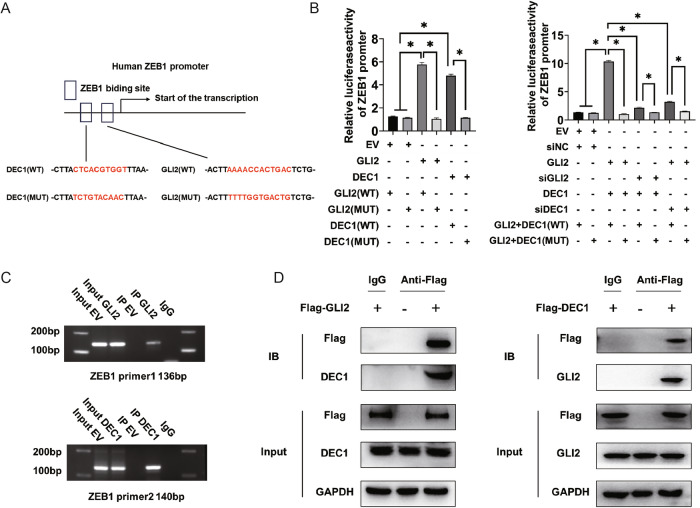
GLI2 and DEC1 form a protein complex that can cooperatively activate ZEB1 expression. **A**, **B** The possible GLI2 and DEC1 binding sites in human ZEB1 promoter. Transcriptional activity of ZEB1 was measured by the luciferase reporter system. Reporter activity is reported as the fold activation relative to Renilla luciferase activity. **C** ZEB1 promoter binding sites with GLI2 and DEC1 were validated by ChIP. **D** Co-IP of GLI2 and DEC1. Flag-tagged GLI2 or DEC1 transcripts were transfected into MKN-45 cells. *P < 0.05.

Based on the finding that both GLI2 and DEC1 have the ability to regulate EMT in GC, we hypothesized that these transcription factors could form a protein complex to synergistically regulate the expression of the target gene. The potential GLI2 binding site and DEC1 binding site were identified at the ZEB1 promoter, which is located upstream of the transcription start site (Fig. 8A). We first explored the ability of GLI2 and DEC1 to synergize in luciferase reporter assays. When GLI2 and DEC1 were cotransfected together they synergistically activated the ZEB1 promoter, and this transactivation activity was higher than GLI2 and DEC1 alone (Fig. 8B). In GLI2 and DEC1 mutants, with the exception of GLI2 and DEC1 binding sites, the activation of ZEB1 was markedly downregulated (Fig. 8B). Therefore, GLI2 and DEC1 synergistically activate the ZEB1 promoter. ChIP assays verified the affinity of GLI2 and DEC1 to the ZEB1 promoter in MKN-45 cells (Fig. 8C, supplementary Fig. 8), indicating that both GLI2 and DEC1 were essential for facilitating ZEB1 transcription. Immunoprecipitation of ectopically expressed GLI2 or DEC1 in HEK-293 T cells showed that both GLI2 coprecipitated DEC1 and DEC1 coprecipitated GLI2 (Fig. 8D). These results suggest that the interaction between GLI2 and DEC1 synergistically enhanced the transcription of downstream ZEB1.

### GLI2 and DEC1 expression correlates with ZEB1 expression and poor prognosis in GC

**Fig. 9:**
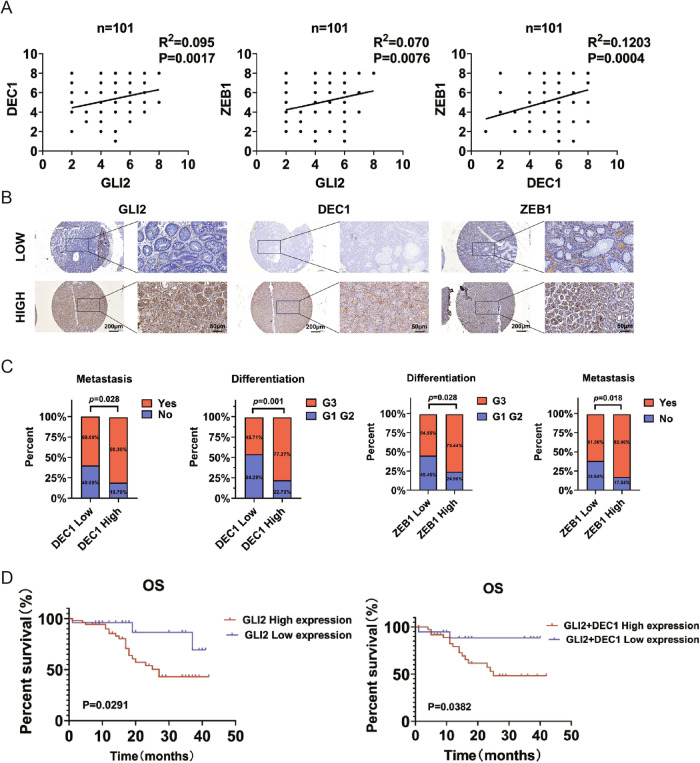
GLI2 and DEC1 expression are correlated with ZEB1 expression and poor prognosis in GC. **A** Correlation analysis of GLI2 with DEC1 and ZEB1. **B** Representative IHC staining for GLI2, DEC1, and ZEB1 in GC tissues. **C** Associations between DEC1 or ZEB1 expression and clinicopathological characteristics of GC. **D** Overall survival was analyzed using Kaplan–Meier curves (log-rank test) in GC patients with GLI2 and DEC1 different expressions.

In order to further explore the clinical application of GLI2, DEC1 and ZEB1, we evaluated their relationship in human GC tissue. Our results showed that there was a significant positive correlation between the expression of GLI2 and DEC1, DEC1 and ZEB1, and GLI2 and ZEB1 (Fig. 9A). The expression levels of DEC1 and ZEB1 in tumor tissues and matched adjacent normal tissues of GC patients were detected by IHC. As shown in Supplementary Fig. 9A, B, the protein levels of DEC1 and ZEB1 in GC tumor tissues were significantly higher than those in adjacent normal tissues. Moreover, our analysis of data from clinical GC patients showed high DEC1 and ZEB1 expression was correlated with differentiation degree and lymph node metastasis (Fig. 9C). Kaplan–Meier survival analysis showed that patients with high GLI2 IHC scores had a markedly shorter OS than those with low GLI2 IHC scores. We then divided the patients into two groups: high GLI2/high DEC1 IHC scores and low GLI2/low DEC1 IHC scores. As shown in Fig. 9D, patients with high GLI2 and high DEC1 IHC scores in tumor tissue had the shortest OS. These data demonstrate that high GLI2 and DEC1 co-overexpression represent an independent factor for poor prognosis.

## Discussion

GC is a common malignant tumor of the digestive system, and resistance to therapy is responsible for treatment failure in the majority of patients, as the mechanisms of its tumorigenesis and associated chemoresistance are poorly understood. Nowadays, EMT is considered an important driver for the development of therapy resistance and is for this reason attracting increasing interest [26]. Here, we identified that GLI2 is overexpressed in EMT-type GC and that GLI2 plays a key role in regulating the resistance of GC cells to CDDP therapy. Additionally, we discovered that GLI2 can bind to the promoter region of DEC1 and enhance its expression, thereby co-transcriptionally regulating ZEB1 expression. Animal studies have demonstrated that both GLI2 knockdown and GLI2 inhibitors significantly enhance CDDP sensitivity in GC. These findings suggest that clinical combination therapy with GLI2 inhibitors may be a novel option in GC.

Conventional chemotherapeutic drugs against GC currently have a bottleneck in effectiveness. GLI proteins are downstream regulatory factors of the Hh signaling pathway. Abnormal expression of GLI2 promotes carcinogenesis by enhancing cell growth and regulating stem cell self-renewal, making it a potential therapeutic target for treating cancer or increasing the effectiveness of chemotherapy [8, 27]. Through an analysis using both the TCGA database and our own collected samples, we observed elevated levels of GLI2 expression in GC tissues that were significantly associated with the patient’s differentiation degree, pathological grade, lymph node metastasis, and tumor size. GC is usually an adenocarcinoma. However, it is known for its large differences between subtypes. According to several genetic, epigenetic, and functional parameters, the ACRG classification categorizes GC into the following molecular subtypes: microsatellite unstable (MSI), EMT, microsatellite- stable/TP53- (MSS/TP53-), and MSS/TP53+. EMT has the worst prognosis [28]. Based on the ACRG dataset, the EMT subtype was observed in which GLI2 was significantly upregulated compared to the other three subtypes. Subsequently, the hypothesis that GLI2 induces EMT to promote migration and CDDP resistance of GC cells was confirmed. Correlation analyses were conducted between the expressions of markers associated with cell proliferation and EMT and those for GLI2 expressions in clinical tissue specimens from GC patients. They showed positive correlations between GLI2 expressions and Ki67 (cell proliferation marker) as well as N-cadherin (EMT marker).

GLI2 can regulate the tolerance of cancer cells to anticancer agents. For example, GLI2 levels are increased in cells isolated from chemoresistant pancreatic cancer cells [29, 30]. Accordingly, inhibition of GLI2 re-sensitizes to chemotherapy in hepatocellular carcinoma [30, 31] and lung cancer [32, 33]. Importantly, GLI2 inhibitors effectively suppressed the growth of Smoothened (SMO) inhibitor-resistant hedgehog-driven cancer models [34]. Most efforts have typically focused on targeting GLI inhibition through the canonical Hh pathway and targeting upstream regulators such as SMO. However, clinical trials have failed in most solid tumors, including GC, likely due to non-canonical activation of GLI2. Therefore, direct targeting of GLI may be a better choice to improve antitumor activity. Small molecule inhibitors targeting the GLI molecule have been developed, such as GANT61 and GANT58, which can disrupt the binding between GLI and DNA. Among them, GANT61 exhibits higher specificity toward GLI and more effectively inhibits the interaction between GLI and DNA [35, 36]. These inhibitors have demonstrated inhibition of tumor cell proliferation in vitro as well as suppression of tumor growth in vivo. For instance, GANT61 has exhibited specific antitumor effects by suppressing GLI expression in models of lung cancer xenografts, acute myeloid leukemia, rhabdomyosarcoma (RMS), neuroblastoma (NB), breast cancer, and pancreatic cancer [37–43]. Our results suggest GLI2 is required for continued cell growth, migration, and EMT in CDDP-treated GC cells. GANT61 can inhibit GLI2 expression in GC cells both in vitro and in vivo, thereby suppressing EMT and CDDP resistance. These results further support that GLI2 can initiate EMT and chemotherapy resistance in GC. In support of our findings, previous findings have reported that EMT plays a pivotal role in conferring chemotherapy resistance in GC [44]. EMT also exerts a critical influence on the malignancy and drug resistance of breast cancer, as its induction is closely linked to radiation resistance in breast tumors [45, 46]. It has been revealed that RHOJ regulates chemotherapy resistance related to EMT by enhancing response to replication stress and activating DNA damage response mechanisms, thereby facilitating the rapid repair of DNA damage caused by chemotherapy agents [47].

As a transcription factor, the action of GLI2 is achieved through the transcription of downstream target genes such as CCND1, SOX2, and Bcl-2, which play roles in cell cycle progression, self-renewal, and anti-apoptosis [30, 48, 49]. In our previous studies, we have shown that differentiated embryonic chondrocyte-expressed gene 1 (DEC1) (also known as BHLHE40/Bhlhb2/Stra13/ Sharp2) drives the proliferation, invasion, and metastasis of GC cells [50–53]. GLI1 has been reported to activate the expression of DEC2, the DEC1 homologs, through a GLI binding site in the promoter in pancreatic ductal adenocarcinoma [54]. Through GLI2 target gene screening, we discovered GLI2 elevated expression of DEC1. Our study also illustrated that DEC1 is essential for GLI2-mediated CDDP resistance and EMT. Previous studies have provided valuable insights into the importance of DEC1 transcription factors as key participants in the development and progression of tumors [51, 55, 56]. We have further dissected the molecular mechanisms by which DEC1 is a direct transcriptional target of GLI2, which is necessary for the induction of ZEB1 expression. ZEB1 drives EMT and confers chemotherapeutic resistance in cancer cells [57, 58]. Our results found that concomitant binding of both GLI2 and DEC1 to the ZEB1 promoter was necessary for efficient transcriptional promotion. We undertook in vivo experiments and demonstrated that the GLI2/DEC1/ZEB1 pathway has a significant effect on GC tumorigenesis. Furthermore, our patient-derived GC tissue array data showed that high GLI2 expression is positively correlated with high levels of DEC1 and ZEB1. More importantly, patients with high protein levels of both GLI2 and DEC1 had a worse prognosis. GLI2 and DEC1 are the potential biomarkers for the diagnosis and prognosis of GC.

## Conclusions

In this study, our data clearly demonstrate that the GLI2/DEC1/ZEB1 signaling axis promoted EMT and enhanced CDDP resistance of GC cells, suggesting that inhibition of GLI2 could enhance therapeutic efficacy in patients with GC. Our research primarily focuses on elucidating the intricate processes underlying EMT in GC cells and confirming GLI2 as the central regulatory molecule governing EMT. We have discovered that EMT actively participates in regulating proliferation, migration as well as chemotherapy resistance in GC cells. Moreover, we have identified the GLI2-DEC1 axis as a key regulator that controls the expression of ZEB1-an essential core molecule involved in orchestrating EMT. Our combined study on the Hh signaling pathway and EMT pathway explores potential molecular mechanisms underlying CDDP resistance, providing insights for clinical treatment with CDDP and the development of Hh-targeted drugs.

## Supplementary information

Supplementary Figure legends

Full and uncropped Western blots

Supplementary Fig. 1

Supplementary Fig. 2

Supplementary Fig. 3

Supplementary Fig. 4

Supplementary Fig. 5

Supplementary Fig. 6

Supplementary Fig. 7

Supplementary Fig. 8

Supplementary Fig. 9

Supplemental Table

## Data Availability

All the data supporting the findings of this study are available from the corresponding author on reasonable request.
