# Supplementary material for: GLI2 inhibits cisplatin sensitivity in gastric cancer through DEC1/ZEB1 mediated EMT
Source: Cell Death Dis. 2025 Mar 25;16(1):204. doi: 10.1038/s41419-025-07564-6 (PMC11937514; doi:10.1038/s41419-025-07564-6)
Supplement: Supplementary file 1 — Supplementary Figure legends [file 41419_2025_7564_MOESM1_ESM.docx]

Supplementary Fig. 1 GLI2 promotes cell proliferation, migration and EMT in GC cells. A. MKN-45 cells and HGC-27 cells were fixed, and nascent DNA was tagged with EdU (red). Nuclei were stained with DAPI (blue), and cells were imaged at 400× magnification. Scale bars are 50 μm. Quantifications are shown in the right panel. B-C. Transwell experiment demonstrated GLI2 mediated cell migration in GC cells. Representative images and quantification of the results are presented. Scale bars: 50µm D-E. Wound healing experiment assays with stably transfected MKN-45 and HGC-27 cells were performed. Quantifications are shown in the right panel. Scale bars: 200µm. F. Western blot analysis investigated the effect of GLI2 on the expression of indicated proteins. G. Western blot analysis investigated the therapeutic effect of GANT61 regulation on GC cells treated with CDDP. H. CCK-8 shows the combine treated data from CDDP and GLI2 knockdown in MKN-28 cells. I. Western blot analysis investigated the effect of GLI2 on the expression of indicated proteins. ****P < 0.0001. ***P < 0.001. **P < 0.01. *P < 0.05.

Supplementary Fig. 2 Inhibited expression of GLI2 sensitizes GC cells to CDDP treatment. A. CCK-8 shows the combine treated data from GANT61 and CDDP in GC cells. B-C. Treatment of MKN-45 cells with CDDP. Cells were then treated with CHX, and GLI2 protein levels were determined by using immunoblotting. D. Representative images for the puromycin experiment and analysis of the effect of GLI2 on general protein synthesis. ****P < 0.0001. ***P < 0.001. **P < 0.01. *P < 0.05.

Supplementary Fig. 3 DEC1 expression is associated with GLI2 in GC cells. A. Volcano plot of MKN-45 EV and GLI2 cell mRNA sequences. B. Correlation analysis in the TCGA database also showed that the expression level of DEC1 was significantly associated with GLI2. C. In tumor tissues from BALB/c Nude mice, DEC1 expression was regulated by GLI2. Scale bars: 50 μm. D. MKN-45 cells with stable DEC1 knockdown or HGC-27 cells with DEC1 overexpression were created. The changes in DEC1 expression were confirmed using western blot. E. The expressions of the transcriptional targets of DEC1( Bcl-2 and MMP9) and Zeb1 (N-cadherin and vimentin). ***P < 0.001. **P < 0.01. *P < 0.05.

Supplementary Fig. 4 Relationship between GLI2, DEC1 and ZEB1 protein expression and sensitivity to CDDP in GC. A. Kaplan-Meier plotter analysis reveals that patients with higher DEC1 and ZEB1 expression have shorter recurrence-free survival (RFS) compared with patients with lower expression. B. Plotted data were mined from the Cancer Therapeutics Response Portal (CTRP, http://www.broadinstitute.org/ctrp/) that contains correlation coefficients between gene expression and drug sensitivity for GC cell lines treated with CDDP. Plotted values are z-scored Pearson’s correlation coefficients. The drug sensitivities were determined using the AUC of the dose-response curves.

Supplementary Fig. 5 Synergistic function of GLI2 and DEC1 on EMT and sensitization to CDDP. A. Western blot analysis investigated the effect of DEC1 and GLI2 on the expression of indicated proteins. B. Transwell experiment demonstrated DEC1 mediated cell migration as GLI2 downstrean in GC cells. Scale bars: 50µm. C-D. Wound healing experiment assays with stably transfected MKN-45 and HGC-27 cells were performed. Scale bars: 200µm. ***P < 0.001. **P < 0.01. *P < 0.05.

Supplementary Fig. 6 DEC1 regulates EMT molecular expressions in GC cells. A qRT-PCR analysis of EMT molecular expressions in MKN-45 cells with or without DEC1 expression. B-C qRT-PCR and western blot analysis ZEB1 expression in MKN-45 and HGC-27 cells with siZEB1. ****P < 0.0001. ***P < 0.001. **P < 0.01. *P < 0.05.

Supplementary Fig. 7 Association of elevated GLI2 expression with CDDP resistance. A. The IC50 values for CDDP in MKN-45 and CDDP resistant MKN-45 R cells. B. Multidrug resistance-associated proteins1 (MRP1) and GLI2 expression was determined by western blot assay. C. Protein levels of GLI2, DEC1, ZEB1 and EMT markers in CDDP resistant and parental MKN-45 cell lines. ****P < 0.0001. ***P < 0.001. **P < 0.01. *P < 0.05.

Supplementary Fig. 8 GLI2 overexpression transactivates DEC1 expression as well as promotes changes in the histone enrichment of the acH3/H4 and H3K4Me3 at DEC1 gene promoter sequences in MKN45 cells. A. Increasing of the enrichment histone code acH3/H4 and H3K4Me3 on DEC1 gene promoter. B-C. ChIP-qPCR analysis at DEC1 and ZEB1 with GLI2 overexpression. Binding to GAPDH and distant region (site3) that is not connected to putative binding sites of either GLI2 or DEC1 served as negative controls. D. Mutation frequency of GLI2 in GC patients. ***P < 0.001. **P < 0.01. *P < 0.05.

Supplementary Fig. 9 The expressions of DEC1 and ZEB1 in adjacent normal tissues and GC tissues. A. IHC staining and H-score of DEC1 in adjacent normal tissues and GC tissues (n=101). Scale bars: 100µm (inset: 50µm). B IHC staining and H-score of ZEB1 in adjacent normal tissues and GC tissues (n=101). Scale bars: 100µm (inset: 50µm).
