# Supplementary material for: GLI2 inhibits cisplatin sensitivity in gastric cancer through DEC1/ZEB1 mediated EMT
Source: Cell Death Dis. 2025 Mar 25;16(1):204. doi: 10.1038/s41419-025-07564-6 (PMC11937514; doi:10.1038/s41419-025-07564-6)

Fig 1G

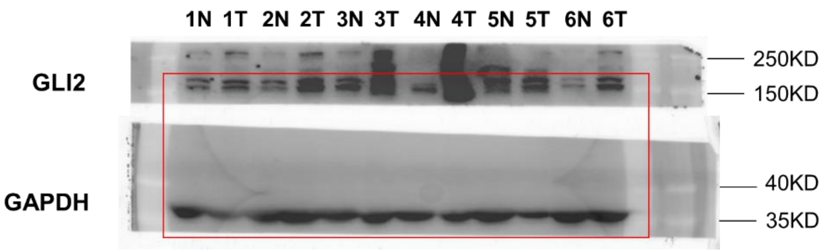

Fig 2A

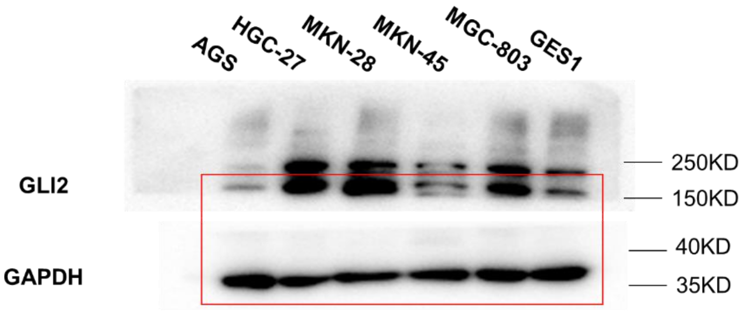

Fig 2B

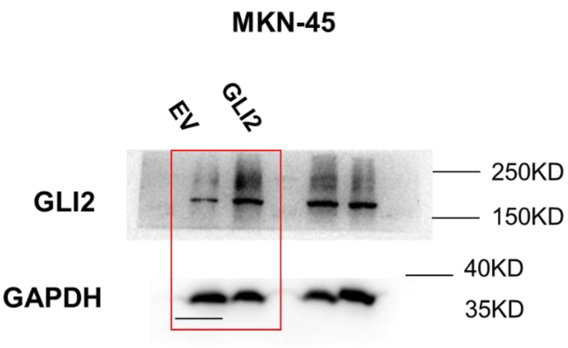

Fig 2C

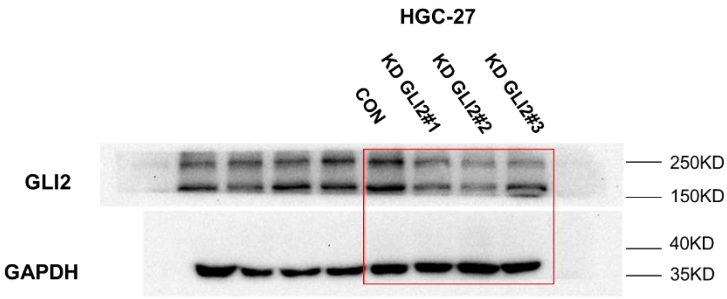

Fig 2F

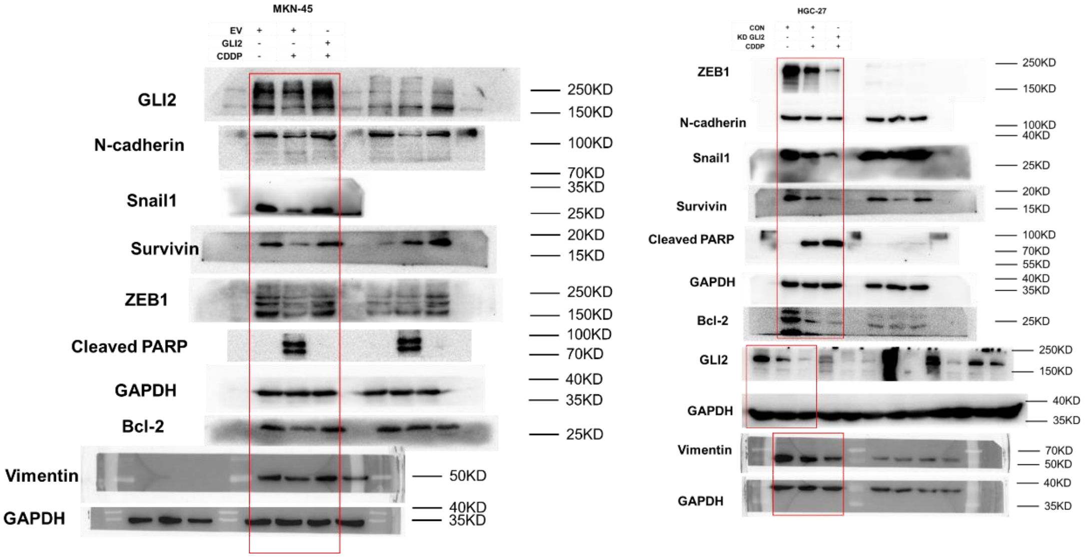
MKN-45
EV + + -
GLI2 - - +
CDDP - + +
GLI2
N-cadherin
Snail1
Survivin
ZEB1
Cleaved PARP
GAPDH
Bcl-2
Vimentin
GAPDH
250KD
150KD
100KD
70KD
35KD
25KD
20KD
15KD
250KD
150KD
100KD
70KD
40KD
35KD
25KD
50KD
40KD
35KD
HGC-27
CON + + -
KD GLI2 - - +
CDDP - + +
ZEB1
N-cadherin
Snail1
Survivin
Cleaved PARP
GAPDH
Bcl-2
GLI2
GAPDH
Vimentin
GAPDH
250KD
150KD
100KD
40KD
25KD
20KD
15KD
100KD
70KD
55KD
40KD
35KD
25KD
250KD
150KD
40KD
35KD
70KD
50KD
40KD
35KD

Fig 4B

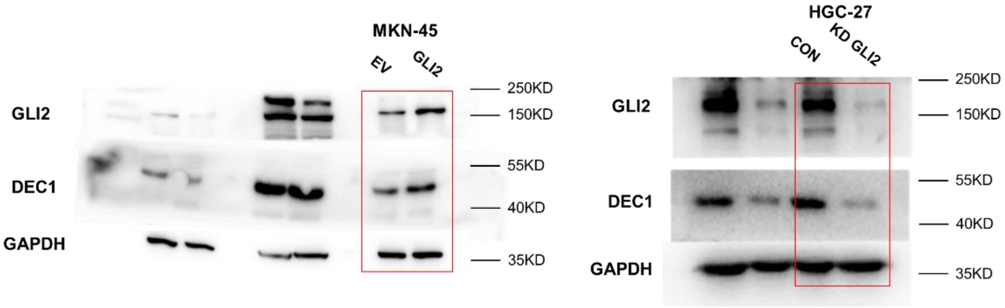
MKN-45
EV
GLI2
GLI2
DEC1
GAPDH
250KD
150KD
55KD
40KD
35KD
HGC-27
CON
KD GLI2
GLI2
DEC1
GAPDH
250KD
150KD
55KD
40KD
35KD

Fig 4D

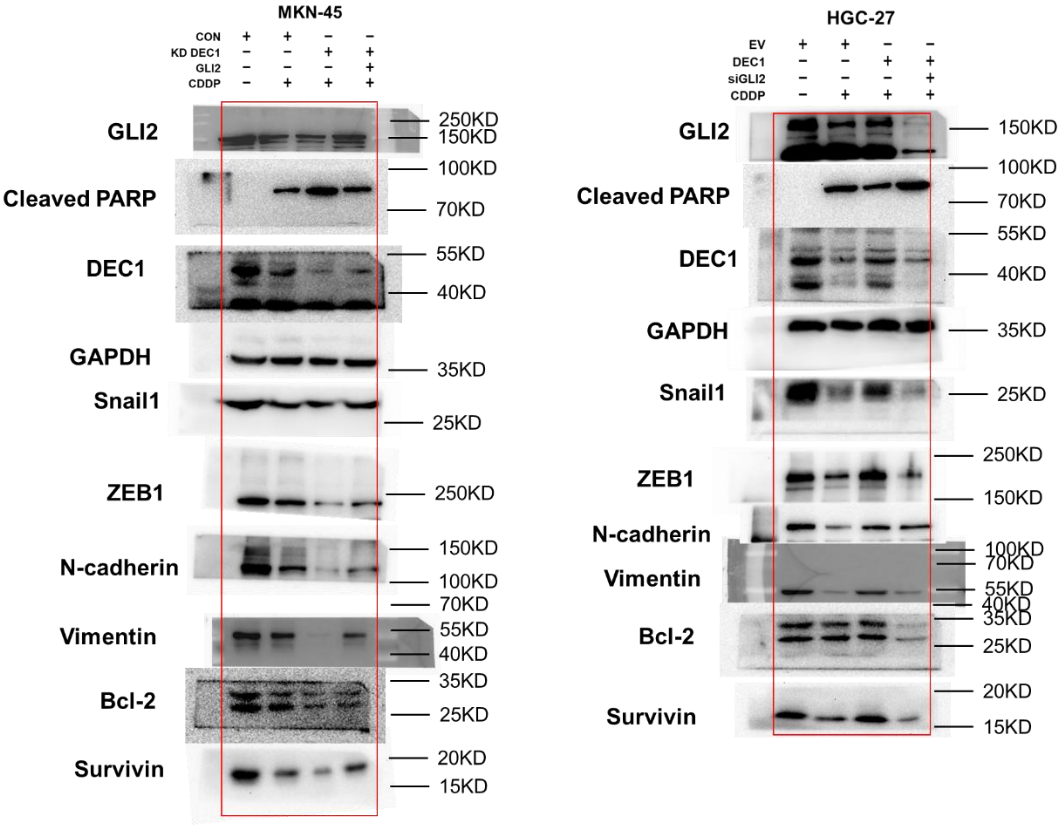

Fig 4E

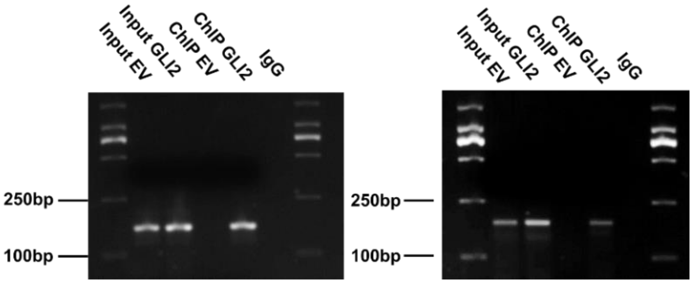

Fig 7A

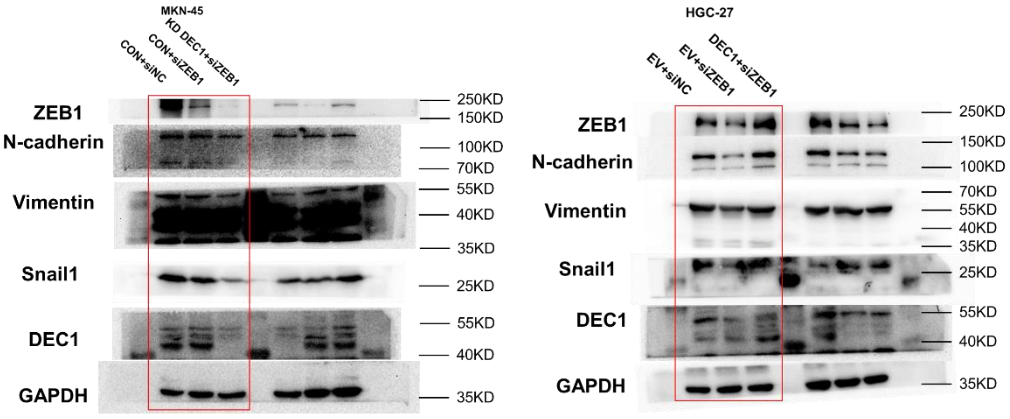

Fig 8C

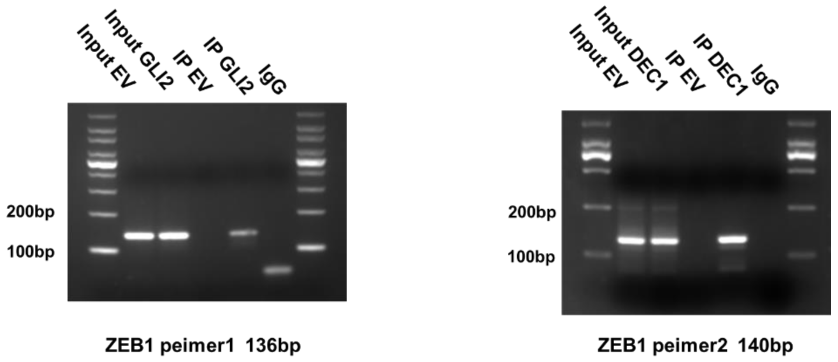

Fig 8D

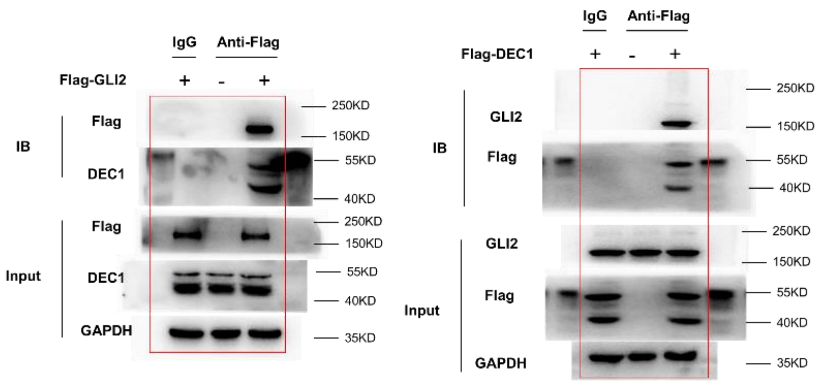

Fig S1F

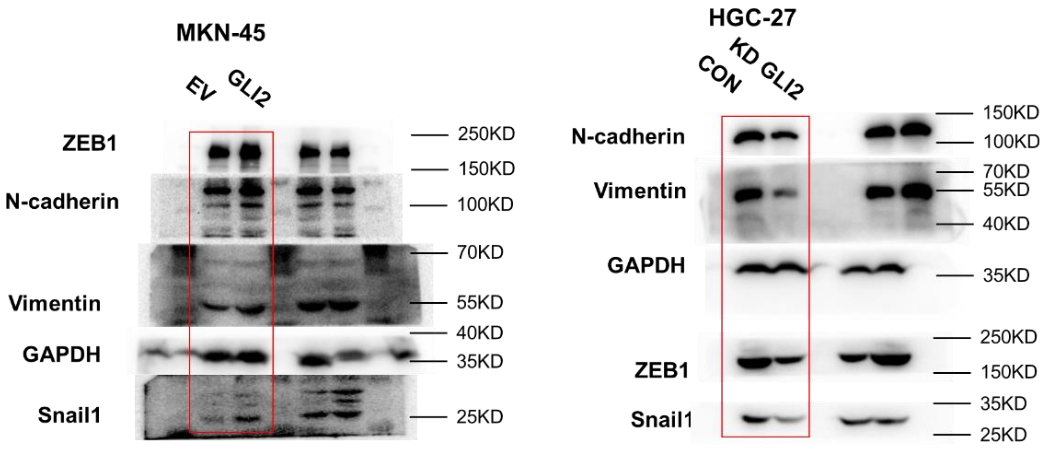

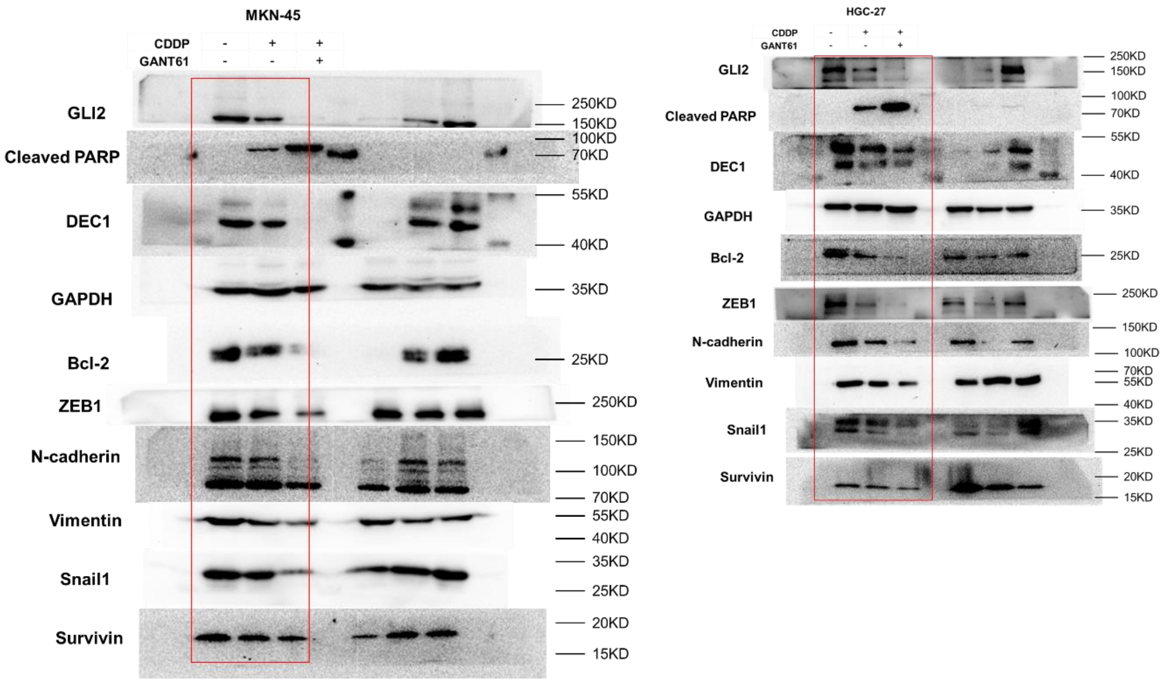

Fig S1G

Fig S1I

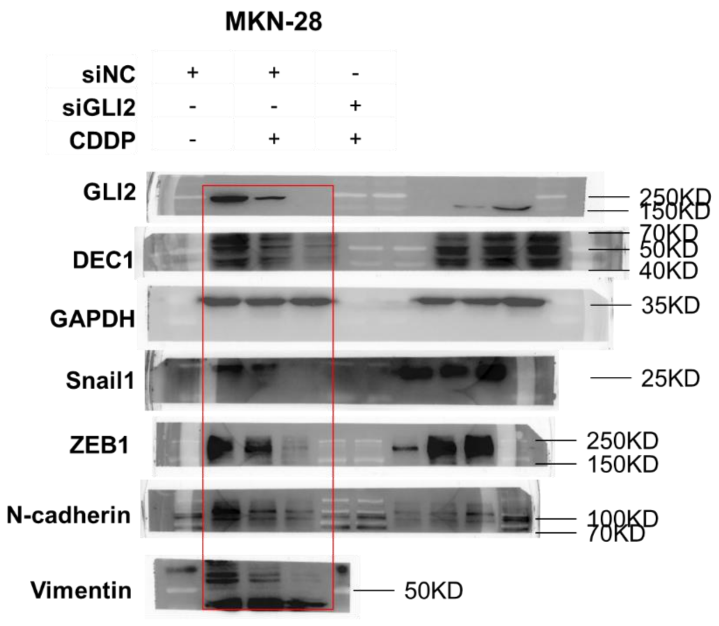

Fig S2B

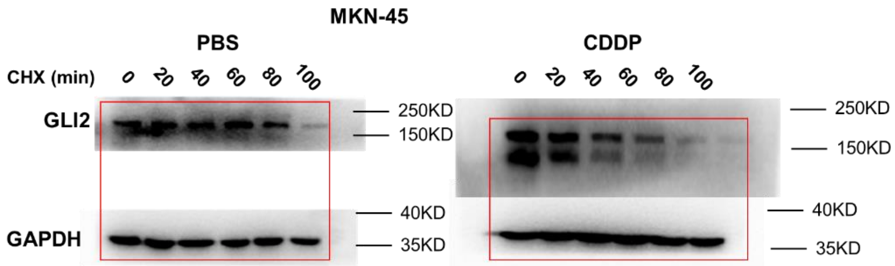

Fig S3D

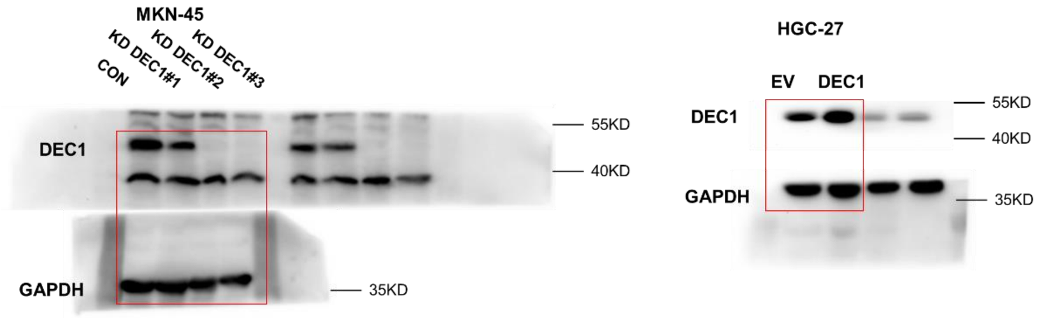

Fig S5A

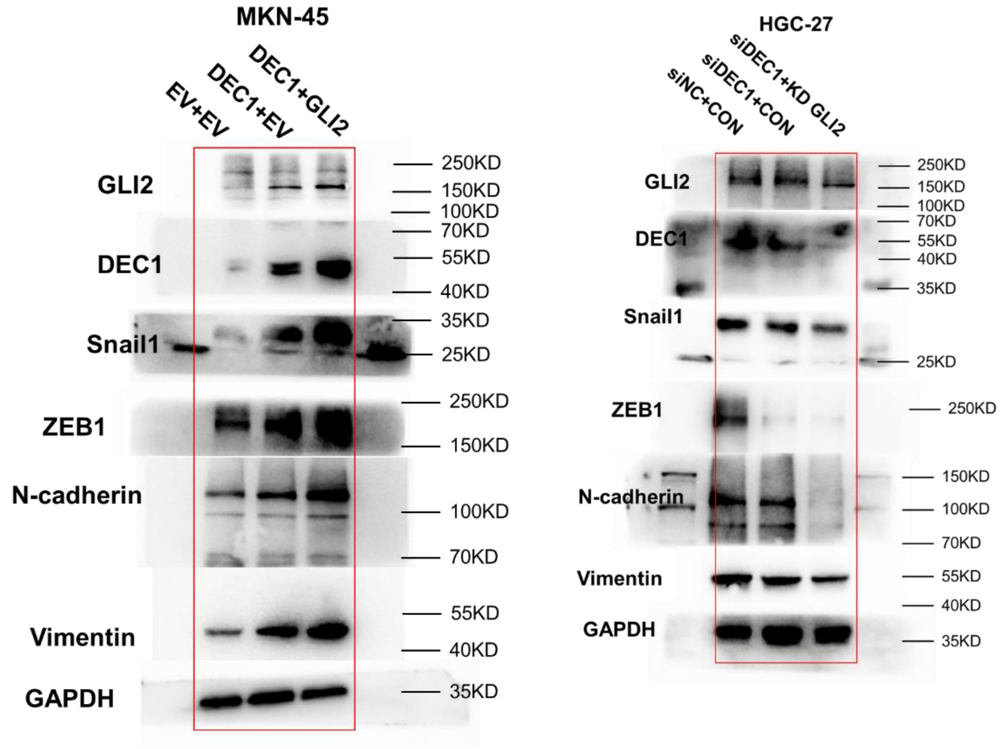

Fig S6C

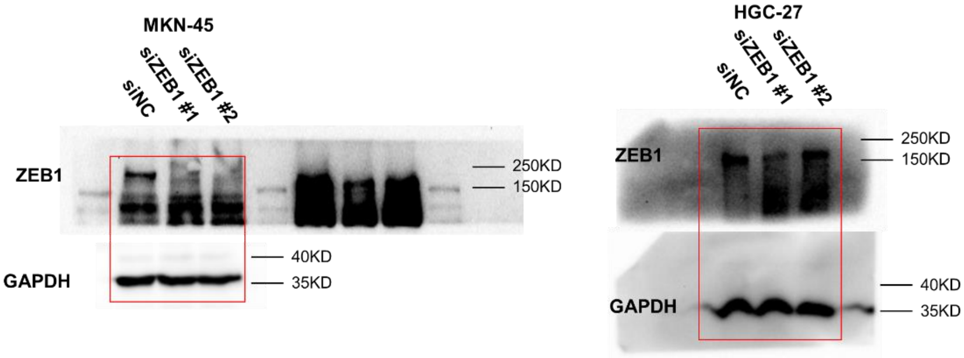

Fig S7B

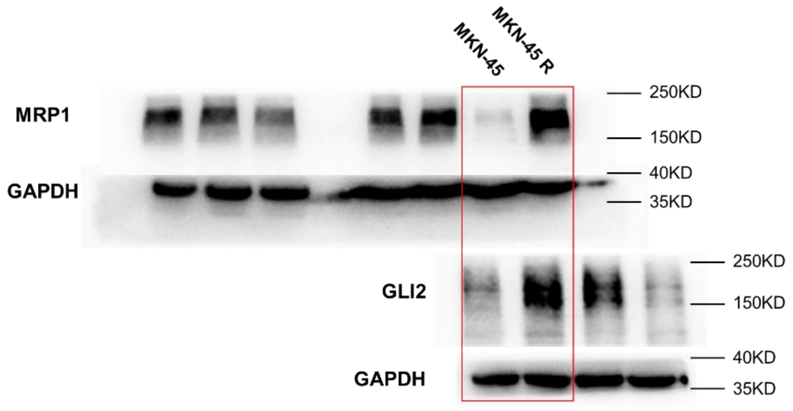

Fig S7C

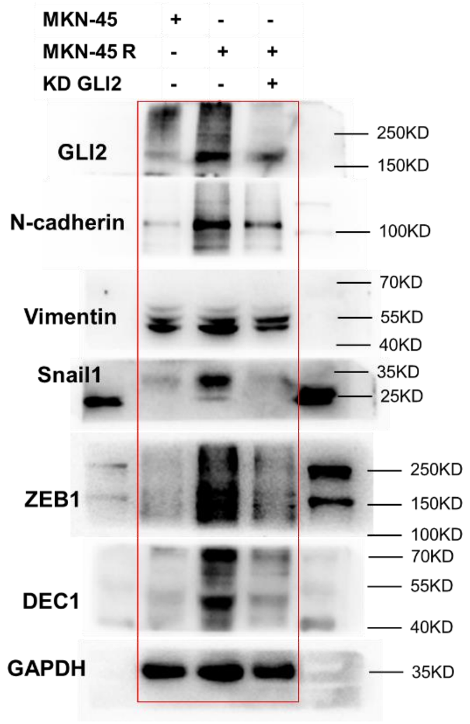

Supplement: Supplementary file 2 — Full and uncropped Western blots [file 41419_2025_7564_MOESM2_ESM.pdf]
