# Supplementary figures and images for: GLI2 inhibits cisplatin sensitivity in gastric cancer through DEC1/ZEB1 mediated EMT
Source: Cell Death Dis. 2025 Mar 25;16(1):204. doi: 10.1038/s41419-025-07564-6 (PMC11937514; doi:10.1038/s41419-025-07564-6)

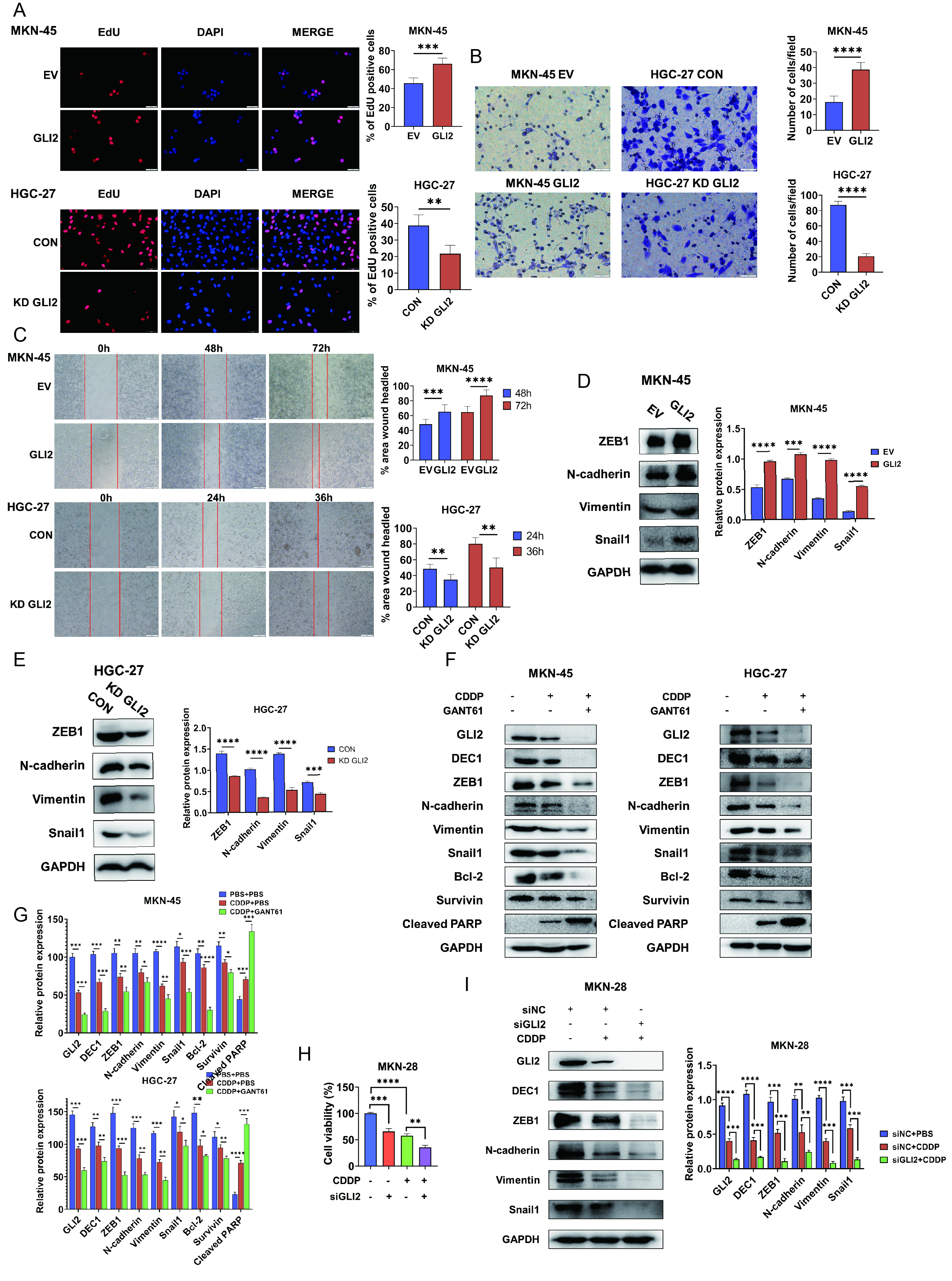

Supplement: Supplementary file 3 — Supplementary Fig. 1 [file 41419_2025_7564_MOESM3_ESM.tif]

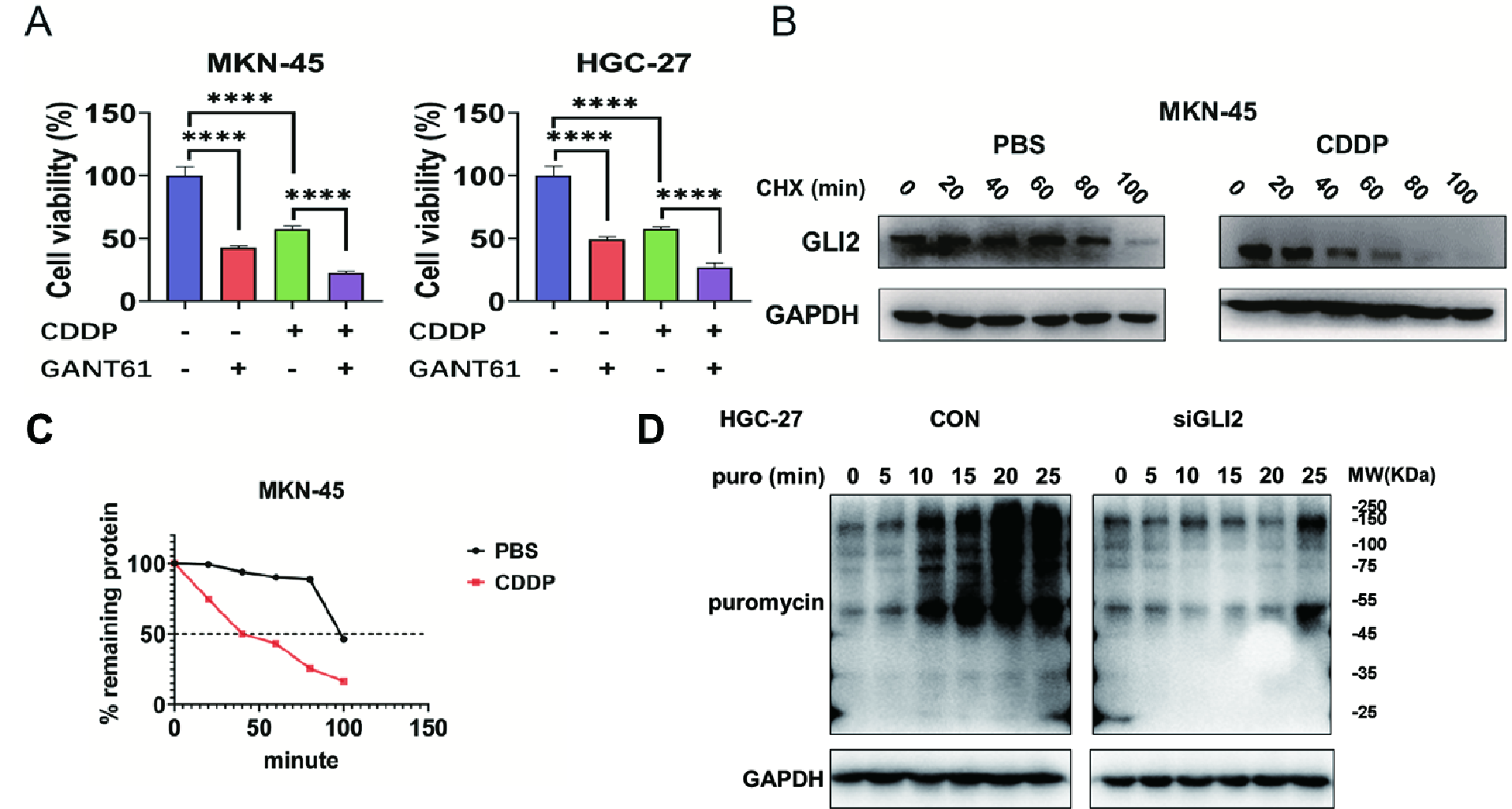

Supplement: Supplementary file 4 — Supplementary Fig. 2 [file 41419_2025_7564_MOESM4_ESM.tif]

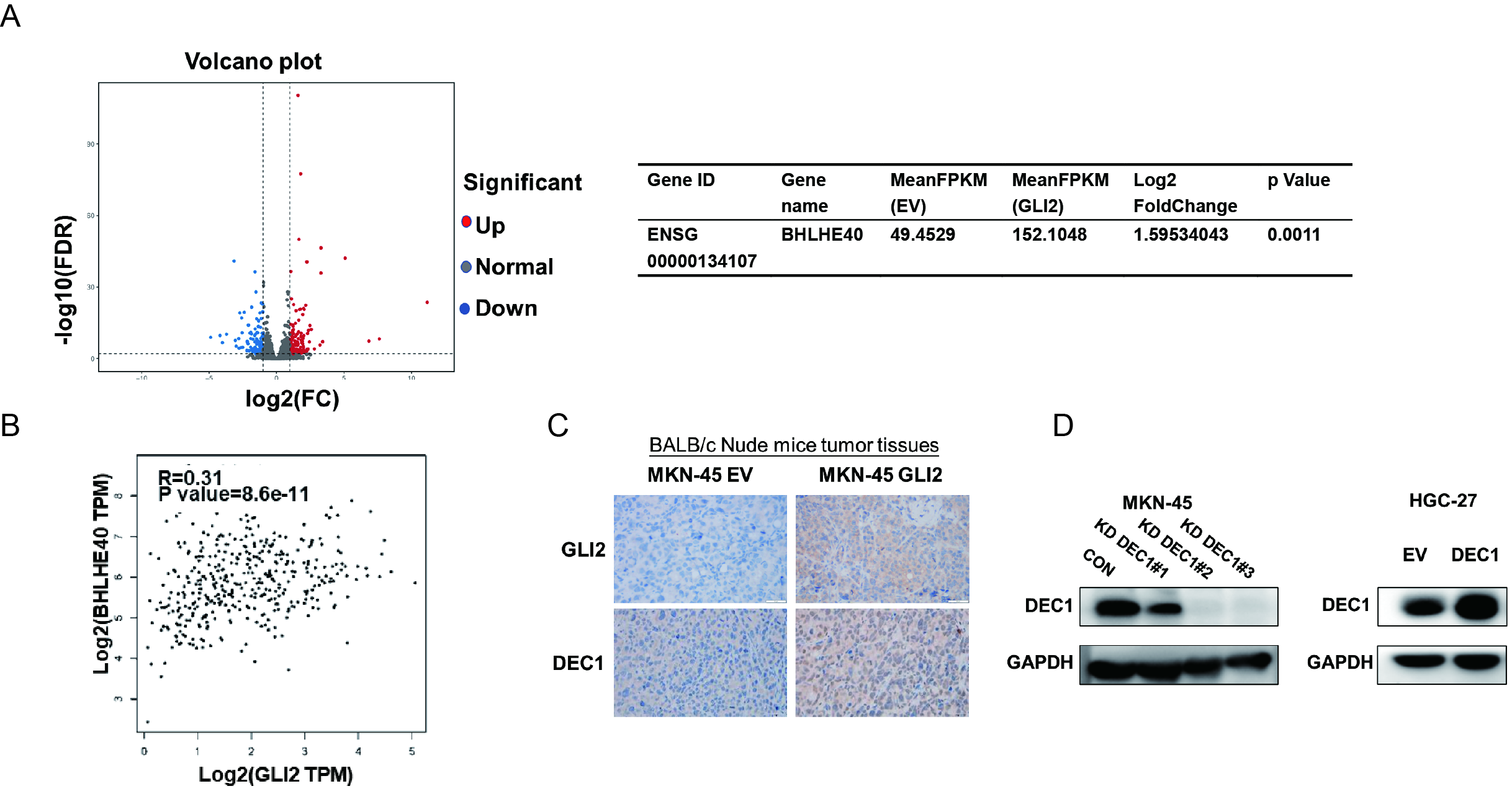

Supplement: Supplementary file 5 — Supplementary Fig. 3 [file 41419_2025_7564_MOESM5_ESM.tif]

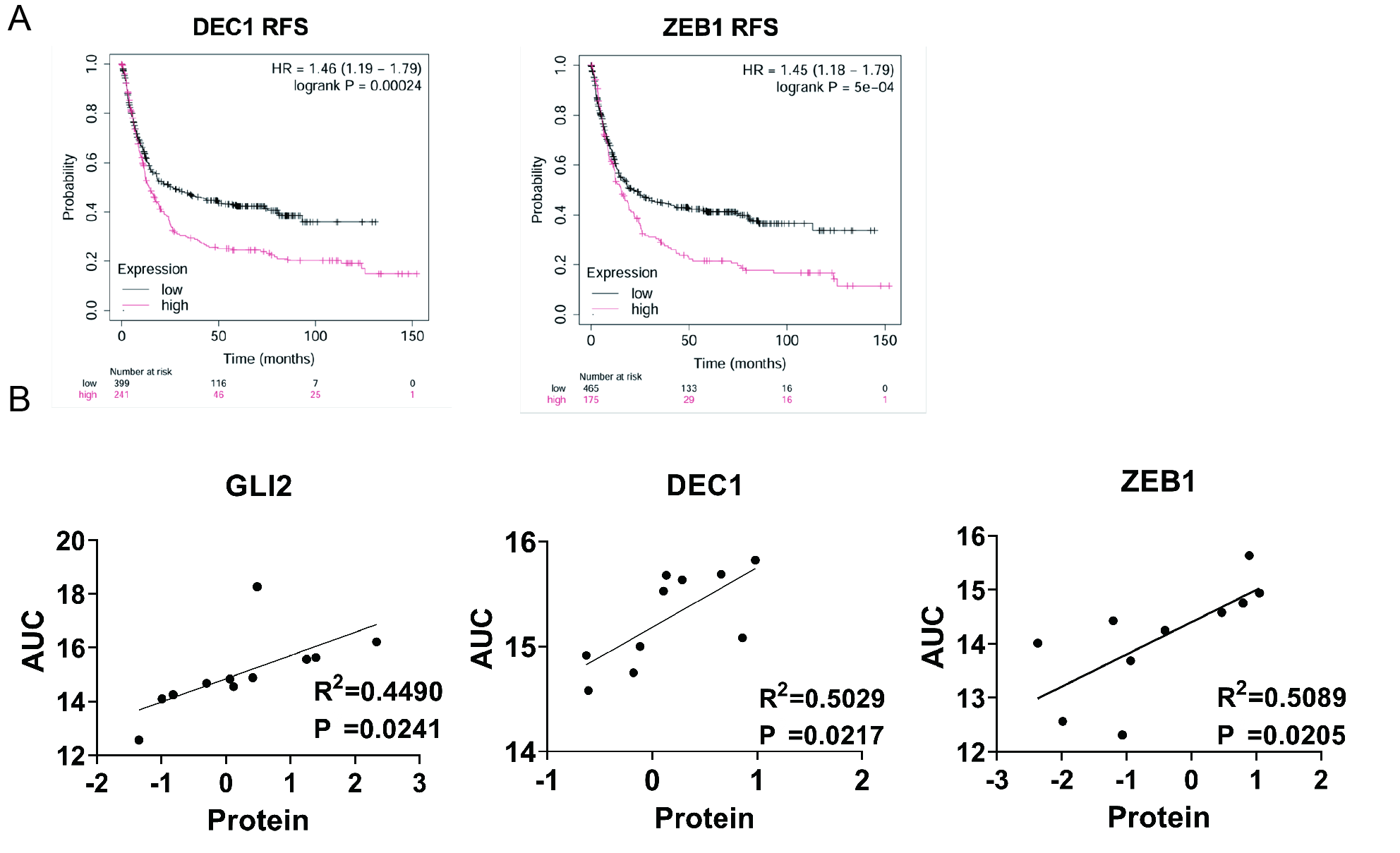

Supplement: Supplementary file 6 — Supplementary Fig. 4 [file 41419_2025_7564_MOESM6_ESM.tif]

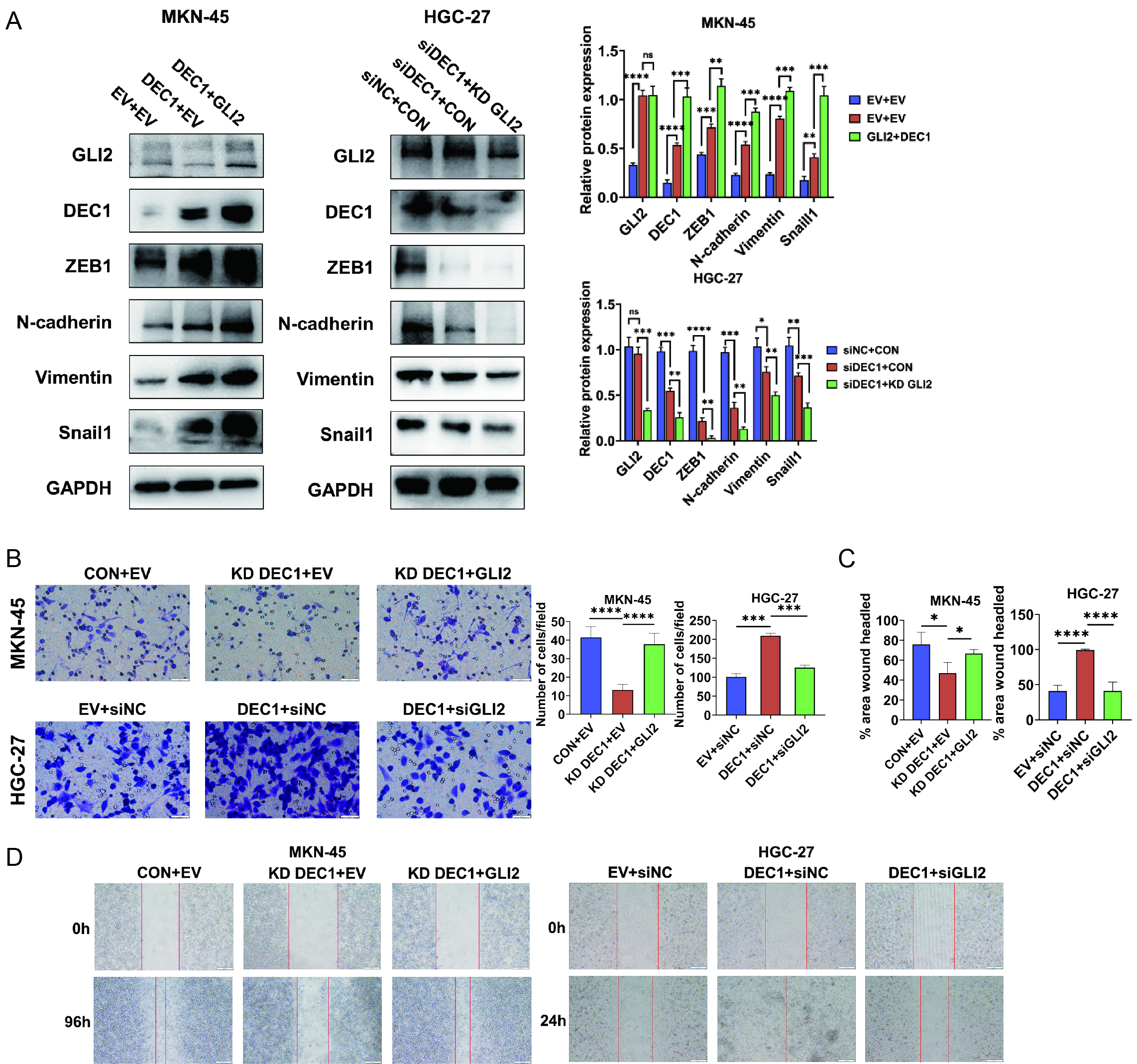

Supplement: Supplementary file 7 — Supplementary Fig. 5 [file 41419_2025_7564_MOESM7_ESM.tif]

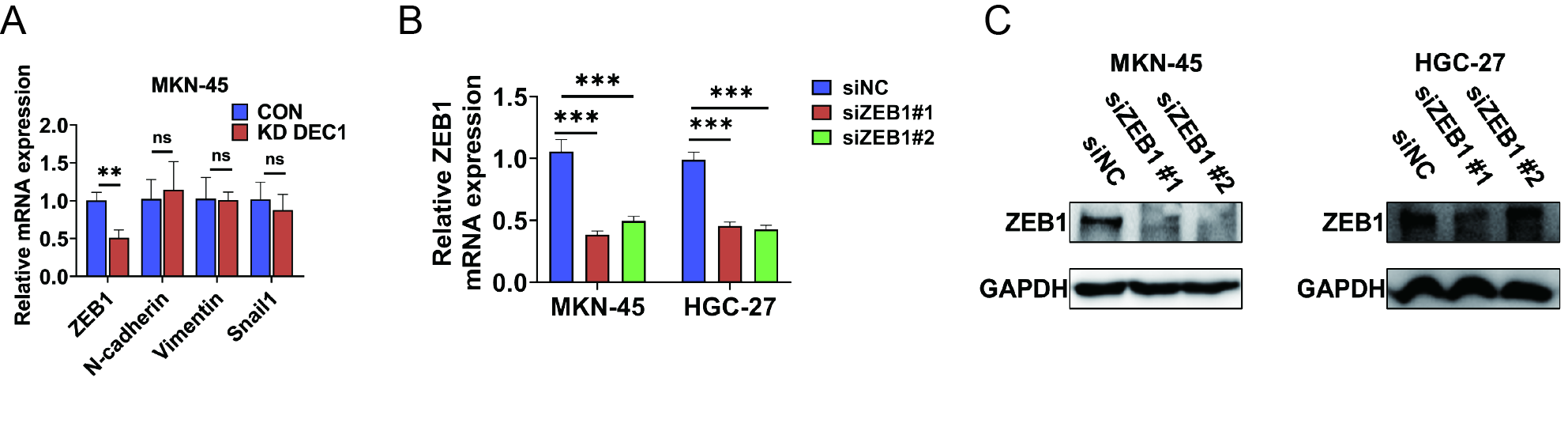

Supplement: Supplementary file 8 — Supplementary Fig. 6 [file 41419_2025_7564_MOESM8_ESM.tif]

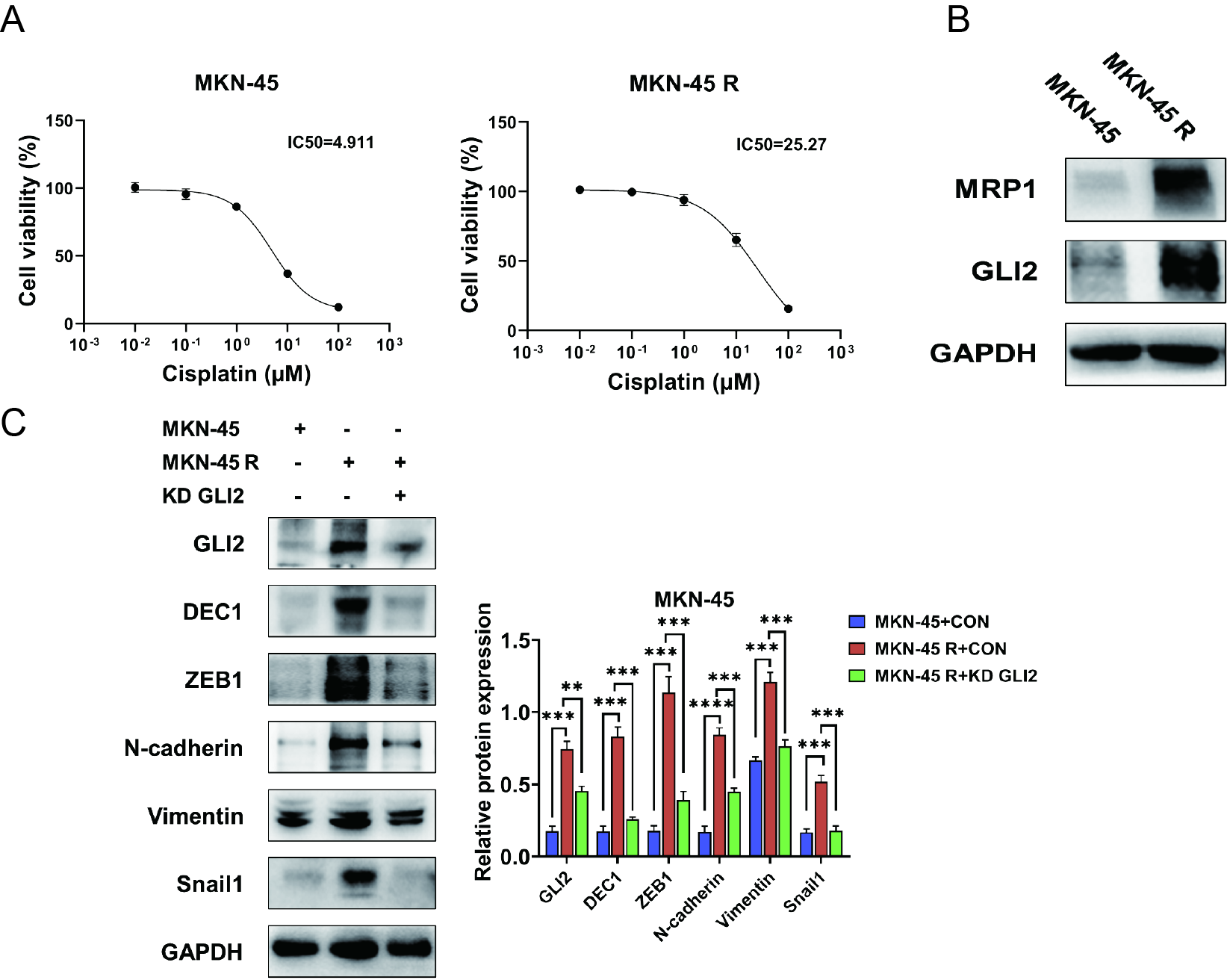

Supplement: Supplementary file 9 — Supplementary Fig. 7 [file 41419_2025_7564_MOESM9_ESM.tif]

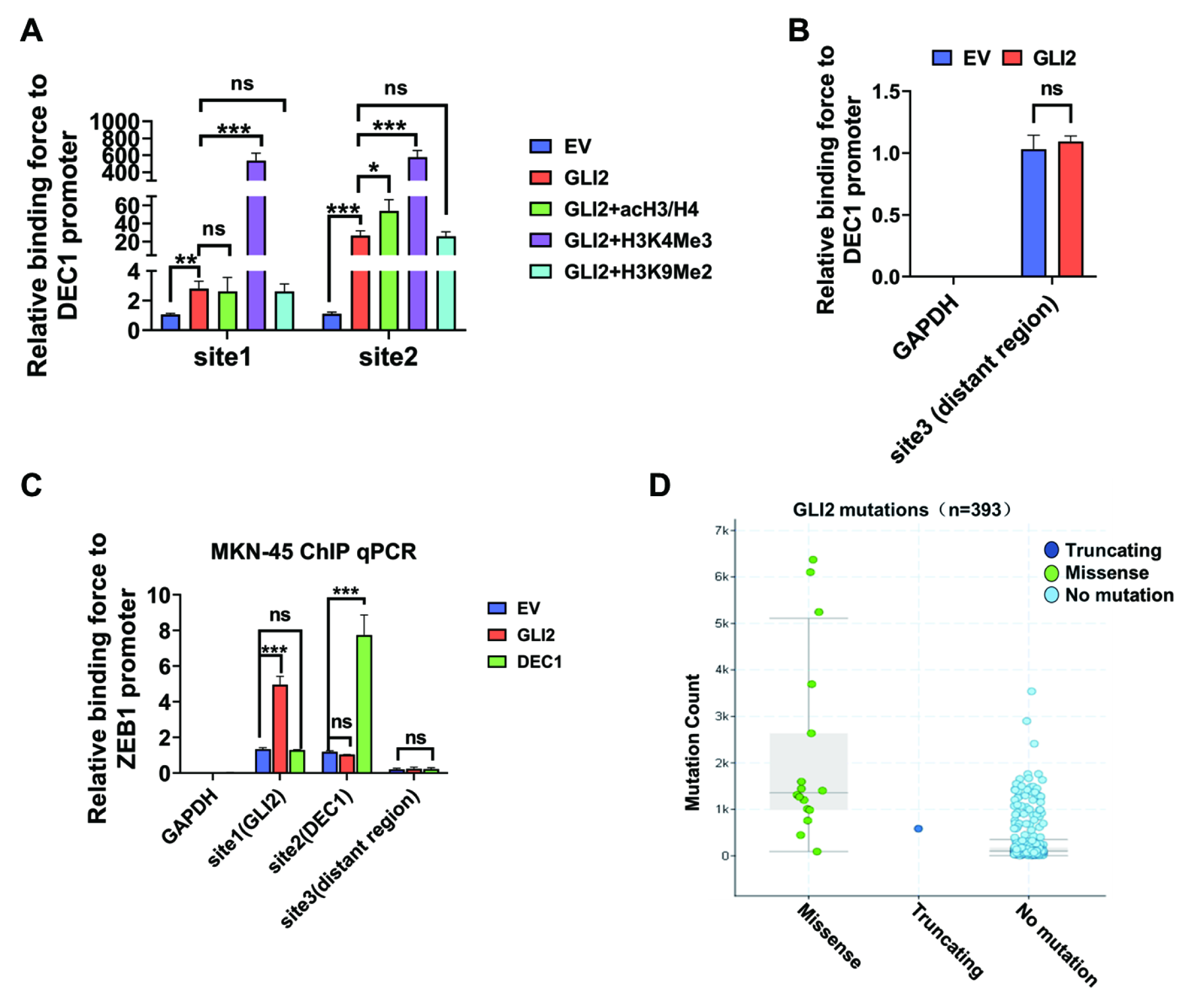

Supplement: Supplementary file 10 — Supplementary Fig. 8 [file 41419_2025_7564_MOESM10_ESM.tif]

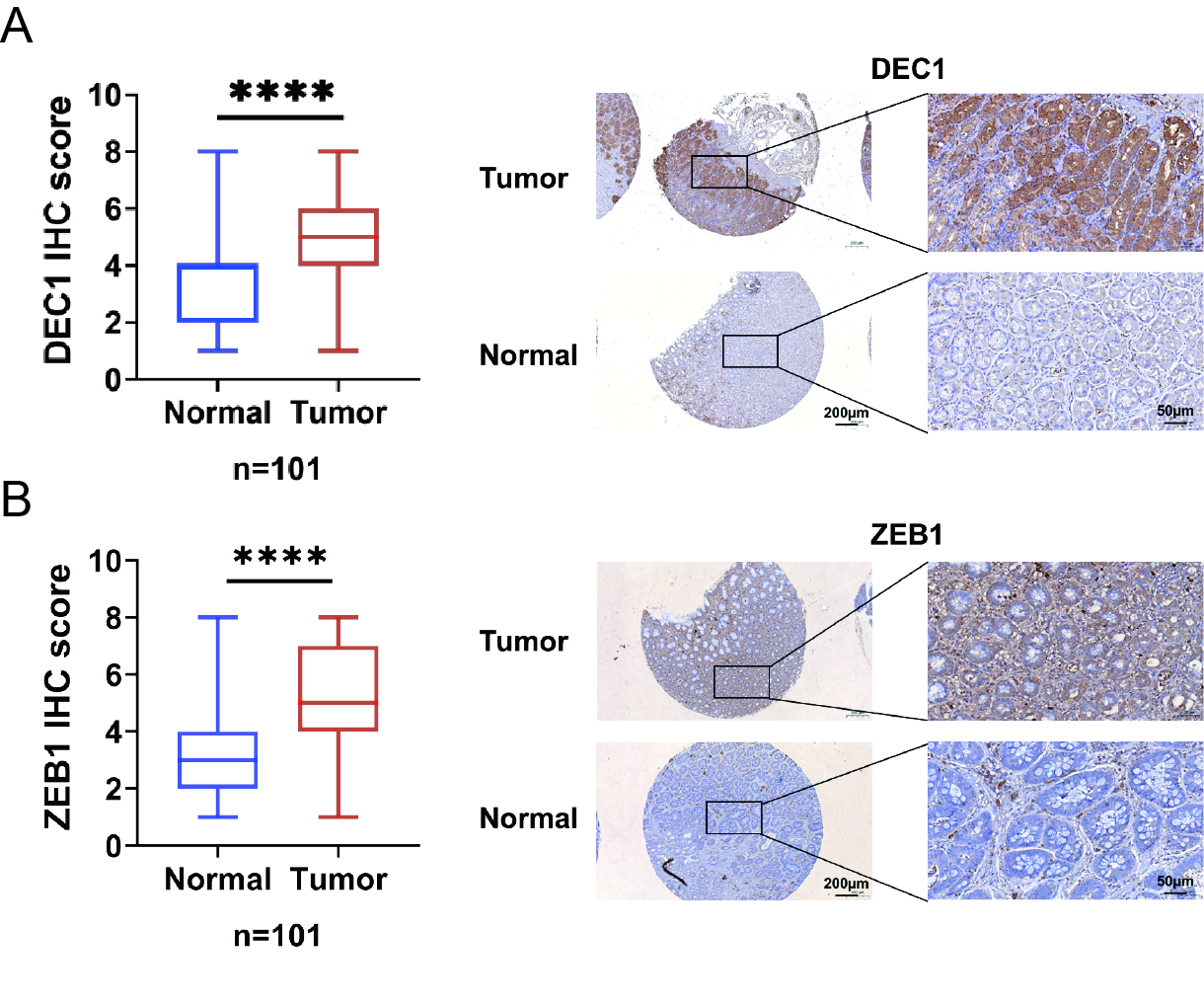

Supplement: Supplementary file 11 — Supplementary Fig. 9 [file 41419_2025_7564_MOESM11_ESM.tif]
