# Supplementary material for: GLI2 inhibits cisplatin sensitivity in gastric cancer through DEC1/ZEB1 mediated EMT
Source: Cell Death Dis. 2025 Mar 25;16(1):204. doi: 10.1038/s41419-025-07564-6 (PMC11937514; doi:10.1038/s41419-025-07564-6)
Supplement: Supplementary file 12 — Supplemental Table [file 41419_2025_7564_MOESM12_ESM.docx]

Supplementary Table 1. PCR primers sequences

| Name | Primer sequences (5' → 3') |
| --- | --- |
| GLI2 (Forward) | CCTGCACGTCAGAGCCATCAAG |
| GLI2 (Reverse) | TCTCCACGCCACTGTCATTGTTG |
| DEC1 (Forward) | GTACCCTGCCCACATGTACC |
| DEC1 (Reverse) | GCTTGGCCAGATACTGAAGC |
| ZEB1 (Forward) | TTACACCTTTGCATACAGAACCC |
| ZEB1 (Reverse) | TTTACGATTACACCCAGACTGC |
| N-cadherin (Forward) | AGCCAACCTTAACTGAGGAGT |
| N-cadherin (Reverse) | GGCAAGTTGATTGGAGGGATG |
| Vimentin (Forward) | AGTCCACTGAGTACCGGAGAC |
| Vimentin (Reverse) | CATTTCACGCATCTGGCGTTC |
| Snail1 (Forward) | TCGGAAGCCTAACTACAGCGA |
| Snail1 (Reverse) | AGATGAGCATTGGCAGCGAG |
| actin (Forward) | TTCCTTCCTGGGCTGGAGTCC |
| actin (Reverse) | TGGCGTACAGGTCTTTGCGG |

Supplementary Table 2. Antibodies utilized in Western Blot

| Antibody | Dilution ratio | Source |
| --- | --- | --- |
| GLI2 | 1:1000 | Abclonal A16864 |
| DEC1 | 1:1000 | Abclonal A6534 |
| ZEB1 | 1:1000 | Boster A00548-2 |
| N-cadherin | 1:1000 | Abclonal A19083 |
| Vimentin | 1:2000 | Proteintech 10366-1-AP |
| Snail1 | 1:1000 | Proteintech 13099-1-AP |
| Bcl-2 | 1:2000 | Proteintech 68103-1-Ig |
| Survivin | 1:2000 | Proteintech 10508-1-AP |
| Cleaved PARP | 1:1000 | CST 9541S |
| GAPDH | 1:10000 | Proteintech 10494-1-AP |

Supplementary Table 3. ChIP-qPCR primers sequences

| Name | Primer sequences (5' → 3') |
| --- | --- |
| GLI2-DEC1#1 (Forward) | CCCAGGAGACGGGAACTTAC |
| GLI2-DEC1#1 (Reverse) | TTTCCCCGAAGAGGGGGTG |
| GLI2-DEC1#2 (Forward) | CTGATCCCCTAACTGCACCC |
| GLI2-DEC1#2 (Reverse) | TTTCACTGACCTCTGCGCTC |
| GLI2-DEC1#3 (Forward) | CCAAGCTTTGGCTGCCCTAT |
| GLI2-DEC1#3 (Reverse) | ATCCCCACAACTTGCTTGCT |
| ZEB1 site1 (Forward) | TGGGGTACATTGTCATCCTGC |
| ZEB1 site1 (Reverse) | CTGAAGCATGAAATGCGGAGG |
| ZEB1 site2 (Forward) | AACCCGCCTTCATCCAATGT |
| ZEB1 site2 (Reverse) | TGAAGGATGAAATGTCCCAGTGT |
| ZEB1 site3 (Forward) | GATGTCTTAGTAGAGCGTATTA |
| ZEB1 site3 (Reverse) | AGTCCCTGCAATCAGAACTCA |

Supplementary Table 4. Dual luciferase reporter system plasmis

| WT-ZEB1 promoter | ACTGAAGTTTCTTACTCACGTGGTTTAAAATGGAGTTCAAAAGATTGCCATTGAGTTCTGATTGCAGGGACTAACAATGTTAATCTGATAAGGACAGCAAAATCATCAGAATCAGTGTTTGTGATTGTGTTTGAATATGTGGTAACATATGAAGGATATGACATGAAGCTTTGTATCTCCTTTGGCCTTAAGCAAGACCTGTGTGCTGTAAGTGCCATTTCTCAGTATTTTCAAGGCTCTAACCCGCCTTCATCCAATGTGTGGCCTACAATAACTAGCATTTGTTGATTTGTCTCTTGTATCAAAATTCCCAAATAAAACTTAAAACCACTGACTCTGTCAGAGAAACTGAAACACTGATATTCCTTCCCCACTAGGAACAGGAACCACATTTGTCATAGTCACTCTCACATTCCTCACTGCCTAACAGGGTGCCTGGCATAAGTTGGGACAACAGATATTTGTTGAATAAAAATATAATTTGCATGTTTATGGAGCTCAGCTATGTTCTCACTTTTTTTGCTTCTAATTCCAGAATATATGTTAAATGATCTAATAATTTGATTATTTTCTTATAAGTCTTATTAAACACTAGTCATAATAGACACAATAAATTATGCCTTC |
| --- | --- |
| MT-ZEB1 promoter | ACTGAAGTTTCTTATCTGTACAACTTAAAATGGAGTTCAAAAGATTGCCATTGAGTTCTGATTGCAGGGACTAACAATGTTAATCTGATAAGGACAGCAAAATCATCAGAATCAGTGTTTGTGATTGTGTTTGAATATGTGGTAACATATGAAGGATATGACATGAAGCTTTGTATCTCCTTTGGCCTTAAGCAAGACCTGTGTGCTGTAAGTGCCATTTCTCAGTATTTTCAAGGCTCTAACCCGCCTTCATCCAATGTGTGGCCTACAATAACTAGCATTTGTTGATTTGTCTCTTGTATCAAAATTCCCAAATAAAACTTTTTTGGTGACTGTCTGTCAGAGAAACTGAAACACTGATATTCCTTCCCCACTAGGAACAAAGGTTGTGCCCGTCATAGTCACTTCTGTGCCTTTCACTGCCTAACAGGGTGCCTGGCATAAGTTGGGACAACAGATATTTGTTGAATAAAAATATAATTTGCATGTTTATGGAGCTCAGCTATGTTCTCACTTTTTTTGCTTCTAATTCCAGAATATATGTTAAATGATCTAATAATTTGATTATTTTCTTATAAGTCTTATTAAACACTAGTCATAATAGACACAATAAATTATGCCTTC |
